# SGLT-2 Inhibitors in Cancer Treatment—Mechanisms of Action and Emerging New Perspectives

**DOI:** 10.3390/cancers14235811

**Published:** 2022-11-25

**Authors:** Mieczysław Dutka, Rafał Bobiński, Tomasz Francuz, Wojciech Garczorz, Karolina Zimmer, Tomasz Ilczak, Michał Ćwiertnia, Maciej B. Hajduga

**Affiliations:** 1 Department of Biochemistry and Molecular Biology, Faculty of Health Sciences, University of Bielsko-Biala, 43-309 Bielsko-Biała, Poland; 2 Department of Biochemistry, Medical University of Silesia, 40-055 Katowice, Poland; 3 Department of Emergency Medicine, Faculty of Health Sciences, University of Bielsko-Biala, 43-309 Bielsko-Biała, Poland

**Keywords:** SGLT-2 inhibitors, empagliflozin, canagliflozin, dapagliflozin, anti-cancer therapy

## Abstract

**Simple Summary:**

Despite significant advances in the treatment of cancer, it remains a major cause of death worldwide and new treatment options are constantly being sought. An interesting new group of antidiabetic drugs, called sodium-glucose cotransporter 2 inhibitors have beneficial effects in patients with heart failure and slow the progression of renal failure. They have recently been shown to additionally exhibit anticancer effects in certain types of cancer. The mechanism of their anticancer action, however, is not yet fully understood. Research into understanding these mechanisms is ongoing and it is hoped that, in the future, this knowledge may be used to develop new cancer treatments using these drugs. The possibility of using this group of drugs in anticancer treatment seems very attractive, especially as they are already used in the treatment of diabetes and heart failure. This article presents the mechanisms of their anti-cancer action which are so far known.

**Abstract:**

A new group of antidiabetic drugs, sodium-glucose cotransporter 2 inhibitors (SGLT-2 inhibitors), have recently been shown to have anticancer effects and their expression has been confirmed in many cancer cell lines. Given the metabolic reprogramming of these cells in a glucose-based model, the ability of SGLT-2 inhibitors to block the glucose uptake by cancer cells appears to be an attractive therapeutic approach. In addition to tumour cells, SGLT-2s are only found in the proximal tubules in the kidneys. Furthermore, as numerous clinical trials have shown, the use of SGLT-2 inhibitors is well-tolerated and safe in patients with diabetes and/or heart failure. In vitro cell culture studies and preclinical in vivo studies have confirmed that SGLT-2 inhibitors exhibit antiproliferative effects on certain types of cancer. However, the mechanisms of this action remain unclear. Even in those tumour cell types in which SGLT-2 is present, there is sometimes an SGLT-2-independent mechanism of anticancer action of this group of drugs. This article presents the current state of knowledge of the potential mechanisms of the anticancer action of SGLT-2 inhibitors and their possible future application in clinical oncology.

## 1. Introduction

Sodium-glucose co-transporter-2 inhibitors (SGLT-2 inhibitors) are a new group of antidiabetic drugs which are also beneficial in the treatment of cardiovascular diseases in both diabetic and non-diabetic patients. Recently they have additionally been shown to have an anticancer effect. In vitro studies in cell culture have confirmed that SGLT-2 inhibitors exhibit an antiproliferative activity against some types of tumours. This has also been confirmed by preclinical studies in vivo. The anticancer activity of SGLT-2 inhibitors has been demonstrated in such cancers as liver, pancreatic, prostate, bowel, lung and breast cancer [1,2,3,4,5,6,7,8,9,10,11]. However, the mechanisms of such action remain unclear. Some types of cancer cells express sodium-glucose co-transporter-2 (SGLT-2). However, even in these types of cancer cells, it is claimed that the mechanism for the anticancer activity of this group of drugs is independent of SGLT-2 [12]. Various mechanisms of action of SGLT-2 inhibitors are, therefore, being considered.

Previous trials have confirmed beneficial clinical effects of using SGLT-2 inhibitors in cardiovascular diseases [13,14,15,16,17,18,19,20]. Their nephroprotective effect has also been confirmed both in patients with and without diabetes [21,22]. There have been many randomised clinical trials to assess the cardiovascular outcomes of SGLT-2 inhibitors (empagliflozin, canagliflozin and dapagliflozin), including the Canagliflozin Cardiovascular Assessment Study (CANVAS), the Dapagliflozin Effect on Cardiovascular Events Thrombosis in Myocardial Infarction 58 (DECLARE—TIMI 58) and the Empagliflozin Cardiovascular Outcome Event Trial in Type 2 Diabetes Mellitus Patients (EMPA-REG OUTCOME) [13,14,15,16,17,18,19,20,23,24]. These clinical trials have revealed that when SGLT-2 inhibitors were administered to patients there was a significant reduction in CV mortality. In addition, there was a decrease in the number of hospitalisations due to heart failure as well as in all-cause mortality [25,26]. These effects cannot be fully explained solely by the antidiabetic, metabolic and haemodynamic effects of this group of drugs [27]. Currently, the various mechanisms of these antidiabetic drugs are being investigated in order to explain these beneficial mechanisms.

As antidiabetic drugs the action of SGLT-2 inhibitors is to decrease the active reverse transport of glucose by SGLT-2 in the proximal renal tubule. This is associated with Na^+^ transport maintained by active Na^+^ extrusion. Normally, about 180 g/day of glucose is filtered into the primary urine. This glucose is then reabsorbed in the proximal tubule mainly with the involvement of SGLT-2. SGLT2 inhibitors significantly decrease this glucose reabsorption and thus induce glycosuria, lowering plasma glucose in an insulin-independent mechanism [28]. Glucosuria is linked with calorie loss and causes a reduction in body weight [28]. Clinical trials with SGLT2 inhibitors have also revealed a reduction in body weight. For example, in the EMPA-REG OUTCOME study, treatment with empagliflozin led to an average weight reduction of approximately 2 kg [13]. This same effect was also confirmed for canagliflozin [28].

The anticancer activity of SGLT-2 inhibitors is an area, after diabetology, cardiology and nephrology, where this interesting group of drugs could be applied and could bring significant clinical benefits. The interest in this group of drugs in the context of their anticancer activity is largely due to the well-known fact that cancer cells typically switch their metabolism from fatty acid oxidation to glucose utilisation [29]. This is because the cancer cells often contain dysfunctional mitochondria which do not provide enough energy from the metabolism of the fatty acids. The expression of many types of glucose transporters has been confirmed in cancer cells. They help maintain the high concentrations of glucose which are needed to provide energy to these cells with high metabolic activity and to provide substrates for RNA and DNA synthesis which is not ensured by fatty acid oxidation. There are two main types of glucose transporters in mammalian cells. The first group are stereospecific glucose transporter proteins (GLUTs) in which glucose transport is driven by a glucose concentration gradient across the cell membrane. To date, 14 GLUTs have been identified [29,30,31,32,33,34,35]. The second group includes SGLTs, which use a transmembrane sodium ion concentration gradient for glucose transport. This family of GLUTs provides a more efficient transportation of glucose into the cell, even against a glucose concentration gradient. There are two types of SGLTs: SGLT-1 and SGLT-2. SGLT-2 is normally expressed only in the proximal tubules in the kidneys and is responsible for approximately 90% of glucose reabsorption from primary urine [28,36,37,38,39]. SGLT-1 is also expressed in the proximal tubule in the kidneys and is responsible for approximately 10% of glucose reabsorption into the blood from primary urine. Apart from the kidney, SGLT-1 is also found in the intestines, lungs, prostate, uterus, heart, eyes, tongue, liver and pancreas [29,30,40,41,42,43,44,45,46]. Selective SGLT-2 inhibitors, such as empagliflozin, dapagliflozin or canagliflozin, used in the treatment of diabetes, have a unique mechanism of action independent of both the effect on insulin production and the effect on insulin sensitivity. SGLT-2 inhibitors cause glucosuria, natriuresis, and uricosuria. The presence of SGLTs (SGLT-1 and/or SGLT-2) have been confirmed in various types of cancer cells, such as, inter alia, hepatocellular carcinoma (HCC), pancreatic, prostate, bowel, lung and breast cancers, as well as brain, head and neck tumours [1,6,34,40,47,48,49,50,51,52]. It should be emphasised that in addition to the expression of SGLT-2 in cancer cells, there is also an increased expression of various types of stereospecific glucose transporter proteins (GLUTs), reflecting the metabolic reprogramming of these cells. However, there are no data from studies regarding the relative proportion between the expression levels of SGLT-2 and the expression levels of the other GLUTs in the above-mentioned tumour types. The possibility of blocking the glucose uptake by cancer cells through selectively inhibiting SGLT-2 seems to be an attractive therapeutic approach, especially since the previously considered attempts to inhibit GLUTs were not feasible because healthy cells need this group of GLUTs to maintain their biological activity. SGLT-2, however, aside from cancer cells, is expressed only in the proximal tubules of the kidneys. Furthermore, numerous clinical trials with patients suffering from diabetes or heart failure have shown that the use of selective SGLT-2 inhibitors is well tolerated and safe [13,14,15,16,17,18,19] and these drugs can also be used in patients without diabetes [53,54].

## 2. SGLT-2 Inhibitors and Cancer in Clinical Studies

The incidence of cancer was assessed in clinical studies with the use of SGLT-2 inhibitors. Diabetic patients, with some exceptions, have a higher risk of developing various types of cancer compared to those without diabetes [55,56,57,58,59,60,61,62,63]. Moreover, previous experience with the use of antidiabetic drugs has shown that some drugs, such as pioglitazone, may increase the risk of bladder cancer [64,65,66,67,68,69]. The first data from studies with dapagliflozin also suggested a significant increase in the risk of breast cancer in patients treated with this drug. However, later studies showed that this increase in the number of breast cancer diagnoses was due to the presence of a pre-existing breast cancer rather than the real effect of dapagliflozin on the risk of breast cancer [70]. This was also confirmed by other, later analyses comparing the use of all SGLT-2 inhibitors, including dapagliflozin, in women with type 2 diabetes, with the use of dipeptidyl peptidase-4 inhibitors (DPP-4is) [71]. The study included 9938 patients commencing SGLT-2 inhibitors therapy and 36,631 patients commencing DPP-4is therapy, and the mean follow-up was 2.6 years. In this large population-based cohort study it was shown that the use of SGLT-2 inhibitors did not cause any increase in the risk of breast cancer compared to DPP-4is [71]. Some concerns about the potential of SGLT-2 inhibitors increasing the incidence of cancers was reported in rats and mice treated with SGLT-2 inhibitors [72,73,74,75]. This mainly concerned bladder cancer. However, research on rats, which addressed the issue of whether dapagliflozin acted as a bladder cancer promoter/progressor, did not confirm this action. The basic mechanism which was supposed to promote this cancer was increased glycosuria. Nevertheless, it was not confirmed that this mechanism was responsible for bladder cancer in rats and humans [50]. There is no evidence linking dapagliflozin with the development of bladder cancer [50,70]. Meta-analyses of clinical trials using SGLT-2 inhibitors have also been performed to assess the possible impact on both the overall incidence of cancer as well as individual cancer types. One meta-analysis included 27 clinical trials with a total of 27,744 patients using SGLT-2 inhibitors and a comparator group of 20,441 patients [76]. Only clinical trials of more than one year duration were included in this meta-analysis. It was shown that SGLT-2 inhibitors do not increase the overall risk of malignancies. Particular attention was paid to the risk of bladder cancer, because previous meta-analyses suggested there was an increased risk of this cancer during treatment with SGLT-2 inhibitors, in particular with empagliflozin [5]. However, these earlier analyses did not include two large clinical trials with canagliflozin and dapagliflozin, which had not yet been published [77,78]. These also included short-term studies which are not a reliable source of long-term effects. The later meta-analysis of 27 clinical trials, cited above, showed no significant increase in the risk of bladder cancer in patients treated with SGLT-2 inhibitors [76,78]. In addition, when assessing the risk of breast and kidney cancer during treatment with canagliflozin, no significant increase in the risk of these cancers was found compared to a group treated with other antidiabetic drugs (insulin, metformin, sulfonylurea, pioglitazone) [70,79]. This was also confirmed by a review of quantitive systematic reviews, which attempted to assess the relationship between SGLT-2 inhibitors and the risk of cancer [80]. This review showed that SGLT-2 inhibitor use is not associated with an increased risk of any cancer which was confirmed when SGLT-2 inhibitors were compared with both a placebo and with active comparators (glimepiride, metformin, sitagliptin, saxagliptin, linagliptin, insulin). Moreover, when the individual, most commonly used antidiabetic drugs, such as dapagliflozin, empagliflozin or canagliflozin, were analysed, no relationship between individual SGLT-2i use and the overall risk of any cancer was demonstrated [80].

Additionally, another meta-analysis of the incidence of bladder cancer in patients treated with dapagliflozin, which comprised a group of more than 9000 patients participating in 21 clinical trials, showed no significant difference in the incidence of bladder cancer between the group treated with dapagliflozin and the control group [81]. This meta-analysis did not indicate a causal relationship between bladder cancer and dapagliflozin, nor did it confirm that dapagliflozin significantly increased the incidence of other types of cancer [81]. This was corroborated by another meta-analysis of 46 independent randomised control trials comprising a total of 34,569 patients over an average duration of 61 weeks [5]. The patients in these studies used different SGLT-2 inhibitors and it was shown that the use of SGLT-2 inhibitors was not associated with a significant increase in the risk of any cancer over the course of the study [5]. A significantly increased risk of bladder cancer, however, was found in a subgroup of obese patients. In this meta-analysis, most cases of bladder cancer came from the EMPA-REG OUTCOME study, which used empagliflozin [25]. The authors suggested that this may be related to the specific population of this study including a high proportion of obese patients. Obesity is a risk factor for the development of bladder cancer [82,83,84]. This meta-analysis also showed that the use of canagliflozin is associated with a significant reduction in the risk of gastrointestinal cancer [5]. Furthermore, a later analysis of 20 empagliflozin and placebo-controlled studies found no significant association between empagliflozin and the incidence of bladder cancer [85]. Moreover, a pooled analysis of 15 phase I-III clinical trials with empagliflozin, used at a dose of both 10 and 25 mg daily, showed no association with an increase in the incidence of any type of cancer [86]. This pooled analysis was based on the treatment of more than 15,000 patient years’ exposure to empagliflozin in placebo-controlled trials, including the EMPA-REG OUTCOME trial [86]. A summary of clinical trials assessing the cancer incidence associated with SGLT-2 inhibitor therapy can be found in Table 1.

## 3. SGLT-2 Inhibitors—Anticancer Mechanisms of Action

The metabolic reprogramming of cancer cells involves changes in the influx of various substrates into the cell and changes in their metabolic reactions. This facilitates the high level of growth and proliferation rate required by cancer cells [88,89]. The best known is the reprogramming of cancer cells in terms of glucose transport and metabolism. Generally, this is dependent on metabolic programming which promotes the use of both aerobic and anaerobic glycolysis. Warburg was the first to describe this phenomenon which is now called the “Warburg effect” [90,91]. The switch of cancer cells to a high level of glycolysis is also associated with increased cellular glucose influx [90,91,92,93,94]. There are changes in the expression of GLUTs and key enzymes such as hexokinase 2 or pyruvate kinase M2 (PKM2) [88,89,95,96,97,98,99]. This reprogramming of the glucose metabolism in cancer cells does not only provide these cells with more energy but also promotes the action of numerous oncoproteins thus ensuring the survival and progression of the tumour [88,89].

### 3.1. Inhibition of β-Catenin Action

Canagliflozin Inhibits the Glucose-Influx-Induced Translocation of β-Catenin from the Cytoplasm to the Cell Nucleus and Enhances the Proteasomal Degradation of β-Catenin in HCC.

**Figure 1:**
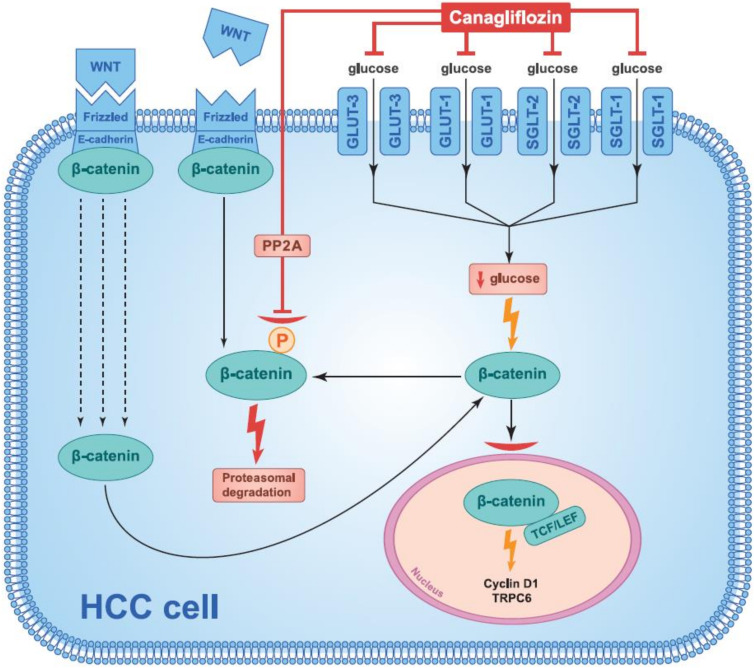
Canagliflozin inhibits β-catenin action and enhances its proteasomal degradation in HCC cells. Canagliflozin blocks glucose influx-induced β-catenin action in HCC cells. Canagliflozin inhibits not only SGLT-2 but also SGLT-1 and other GLUTs, mainly GLUT1, which is overexpressed in HCC cells. It has been confirmed that canagliflozin inhibits the cellular glucose influx in HCC by not only inhibiting SGLT-2, but, to an even greater extent, by inhibiting other GLUTs. When it comes to β-catenin, canagliflozin exerts its anticancer effect on HCC in two ways. Firstly, it inhibits the cellular glucose-influx-induced translocation of β-catenin from the cytoplasm to the cell nucleus. Secondly, canagliflozin enhances the proteasomal degradation of β-catenin by directly inhibiting PP2A activity. The activation of the β-catenin signalling pathway begins with the binding of WNT ligands to Frizzled receptors and LRP-coreceptors, resulting in the dissociation of β-catenin from E-cadherin. After dissociation from E-cadherin, the free β-catenin moves from the cytoplasm to the cell nucleus and there forms a complex with transcriptional co-activators (TCF/LEF). This ultimately leads to the transcription of genes such as CYCLIN D1 and TRPC6, which are involved in cancer development and progression. When WNT is absent or cannot bind to Frizzled receptors, β-catenin phosphorylation occurs. This process ultimately leads to proteasome-mediated degradation of β-catenin. Canagliflozin directly inhibits PP2A activity, which regulates β-catenin phosphorylation. This leads to increased proteasomal degradation of β-catenin. Explanation of abbreviations: SGLT-1: sodium-glucose co-transporter-1; SGLT-2: sodium-glucose co-transporter-2; GLUT1: stereospecific glucose transporter 1; GLUT3: stereospecific glucose transporter 3; PP2A: protein phosphatase 2A; TCF/LEF: T-cell factor/lymphoid enhancer factor; TRPC6: transient receptor potential cation channel.

Changes in HCC are a prominent example of the reprogramming mentioned above. Since the liver is involved in the maintenance of physiological blood glucose levels, a high level of bi-directional glucose transporters, such as GLUT2, is present in normal liver cells [100,101]. Thanks to this, hepatocytes not only take up glucose but also release it outside the cell, thus helping to maintain an appropriate level of glucose homeostasis in the blood. In HCC cells, however, there is a high expression of GLUT1 and GLUT3, which only transport glucose into the cell. Additionally, SGLT-2 expression has been confirmed in HCC cells [102]. Furthermore, it has been confirmed that the cellular influx of glucose into HCC cells enhances the translocation of β-catenin, a transcription factor, from the cytoplasm to the cell nucleus. In vitro studies, assessing the potential anticancer action of SGLT-2 inhibitors in relation to HCC, have shown that canagliflozin blocks the translocation of β-catenin from the cytoplasm to the cell nucleus. This occurs, however, in a mechanism that is at least partially independent of SGLT-2 [89]. Canagliflozin does not only inhibit SGLT-2 but also SGLT-1 and other GLUTs, among these mainly GLUT1, which is overexpressed in HCC cells. Other SGLT-2 inhibitors, such as empagliflozin and dapagliflozin, which are more specific for SGLT-2, have been shown not to interfere with the cellular influx of glucose into HCC. They also have no inhibitory effect on the growth of HCC cells, although such an effect has been shown by specific GLUT1 inhibitors such as phloretin and WZB117. This effect, however, was significantly smaller than with canagliflozin, which indicates that canagliflozin does not only exert its inhibitory effect on HCC growth by inhibiting cellular glucose influx. This was also confirmed in studies using short hairpin ribonucleic acid (shRNA) mediated knockdown of the two most important glucose transporters, GLUT1 and GLUT3 [89] which did not weaken the anticancer effect of canagliflozin. These studies confirmed that the inhibition by canagliflozin of the cellular glucose influx into HCC does not only occur through the inhibition of SGLT-2, but, to an even greater extent, by the inhibition of other GLUTs. [89]. Indeed, studies have confirmed that canagliflozin exerts its anticancer activity on HCC in two ways. Firstly, it inhibits the glucose-influx-induced translocation of β-catenin from the cytoplasm to the cell nucleus. Secondly, it enhances the proteasomal degradation of β-catenin by direct inhibition of protein phosphatase 2A (PP2A) activity [89]. When this degradation occurs the β-catenin can no longer act as a transcriptional factor as described below. The Wnt/β-catenin signalling pathway enhances aerobic glycolysis in cancer cells, increasing the activity of pyruvate carboxylase and the expression of pyruvate dehydrogenase kinase 1 [103,104]. This signalling pathway promotes the metabolic reprogramming of cancer cells, thus increasing their glucose utilisation. The activation of this pathway occurs as a result of increased cellular glucose influx. Genetic studies have confirmed that the excessive activation of the Wnt/β-catenin signalling pathway is a predisposing factor for the development of HCC [89,105]. For this reason, this pathway is seen as an attractive therapeutic target in HCC. Wnt proteins belong to the family of cysteine-rich glycoproteins, which play an important role in the development, formation and growth of cancers. Activation of this signalling pathway begins when Wnt ligands combine with Frizzled receptors and lipoprotein receptor related proteins (LRP) co-receptors, resulting in the separation of β-catenin from E-cadherin. After this separation, this free β-catenin moves from the cytoplasm to the cell nucleus and there forms a complex with transcriptional co-activators. This, ultimately, leads to the transcription of genes such as cyclin D1 and TRPC6, which are involved in the development and progression of cancer [106,107] (Figure 1). In addition, c-Myc, which is the downstream factor of β-catenin, transcriptionally controls numerous key elements responsible for the metabolic reprogramming of cancer cells, such as forkhead transcription factors, pyruvate kinase M2 (PKM2) or GLUT1, among others [89]. When Wnt is missing or unable to bind to Frizzled receptors, β-catenin phosphorylation occurs. This process occurs within a multi-protein complex (destruction complex) and ultimately leads to proteasome-mediated degradation of β-catenin [106,107]. As shown in studies, canagliflozin directly inhibits the activity of PP2A, which regulates the phosphorylation of β-catenin. This leads to an increase in the proteasomal degradation of β-catenin [89].

### 3.2. The Role of AMPK Activation

#### 3.2.1. The Inhibition of Complex I and α Subunit of ATP Synthase F1 in the Mitochondrial Electron Transport Chain

Canagliflozin Inhibits Oxidative Phosphorylation in HCC Cells and Prostate, Lung, Liver and Breast Cancer Cells.

**Figure 2:**
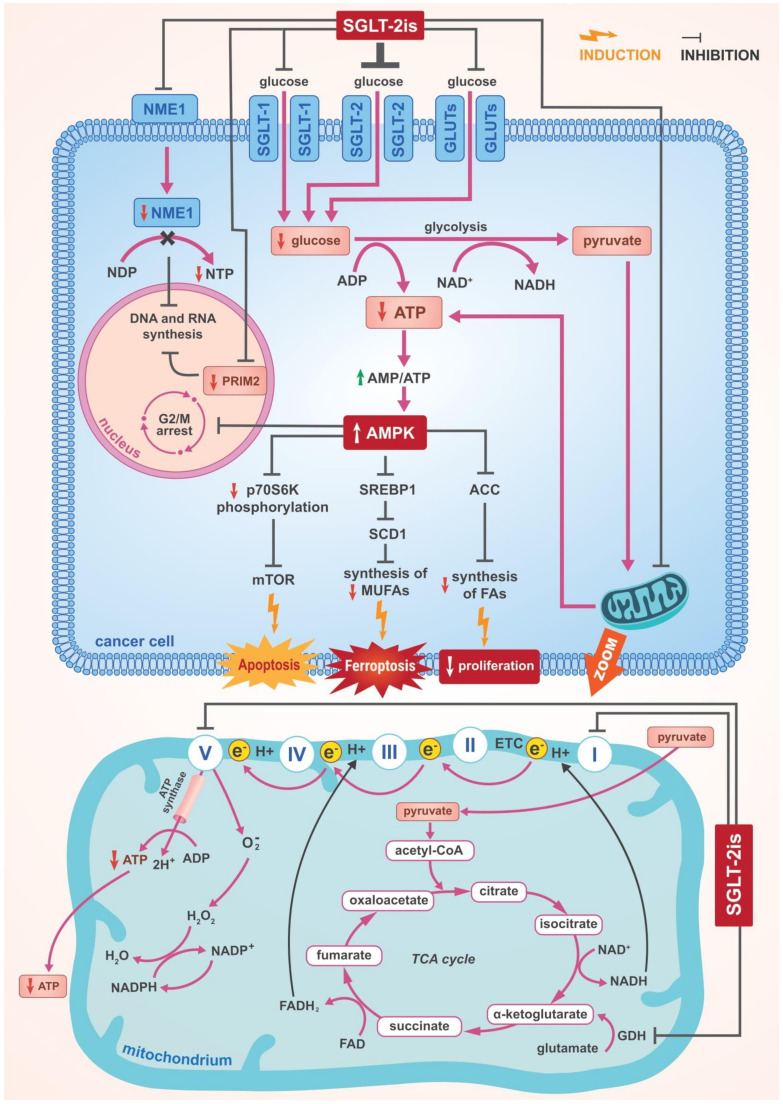
The main mechanisms of anti-cancer action of SGLT-2 inhibitors including the central role of AMPK activation. SGLT-2 inhibitors indirectly activate AMPK in cancer cells. SGLT-2 inhibitors, by inhibiting the oxidative phosphorylation, decrease ATP production and thus increase the AMP/ATP ratio. This leads to AMPK activation. SGLT-2 inhibitors affect oxidative phosphorylation. SGLT-2 inhibitors downregulate the alpha subunit of ATP synthase F1 and inhibit mitochondrial complex-I which leads to a significant decrease in ATP production in cancer cells. This results in an increase in the AMP/ATP ratio, which in turn causes a rapid and significant increase in AMPK activity. Another mechanism of action of SGLT-2 inhibitors, related to their effect on glutamine metabolism, also contributes to the increase in the AMP/ATP ratio. Glutamine is converted to glutamate, which is the carbon source for the TCA cycle. SGLT-2 inhibitors inhibit GDH activity, resulting in a significant decrease in intracellular alpha-ketoglutarate concentration. A fall in alpha-ketoglutarate concentration results in a reduction in its incorporation into the TCA cycle and consequently a decrease in the production of NADH and NADPH. Reduced NADH concentration results in reduced ATP production leading to AMPK activation, while reduced NADPH concentration results in impaired mitochondrial antioxidant defence. The SGLT-2 inhibitor-induced increase in AMPK activity results in multidirectional consequences in cancer cells. One of them is a significant increase in ACC phosphorylation and another one is inhibition of mTOR. ACC phosphorylation leads to its inhibition and thus SGLT-2 inhibitors rapidly inhibit fatty acid synthesis in cancer cells, resulting in the suppression of proliferation of these cells. Inhibition of mTOR is thought to be at least partly responsible for the suppression of proliferation and for induction of apoptosis in cancer cells. In addition, SGLT-2 inhibitor-induced AMPK activation results in the inhibition of SREBP1. The activation of AMPK inhibits SREBP1, which leads to the inhibition of SCD1. This results in the suppression of MUFA production and the dominance of PUFAs, which enhance lipid peroxidation and induce ferroptosis. SGLT-2 inhibitor-induced AMPK activation also leads to inhibition of the cancer cell cycle in the G2/M phase. Additionally, SGLT-2 inhibitors downregulate NME1 and upregulate NDP in cancer cells. This downregulation of NME1 inhibits the synthesis of RNA and DNA. Moreover, SGLT-2 inhibitors downregulate expression of PRIM2, thus interfering with DNA replication and mRNA transcription. Explanation of abbreviations: SGLT-2 inhibitors: Sodium-glucose co-transporter-2 inhibitors; SGLT-1: sodium-glucose co-transporter-1; SGLT-2: SGLT-2: sodium-glucose co-transporter-2; GLUTs: stereospecific glucose transporters; AMPK: adenosine monophosphate-activated proteine kinase; AMP: adenosine monophosphate; ADP: adenosine diphosphate; ATP: adenosine triphosphate; TCA: tricarboxylic acid; GDH: glutamate dehydrogenase; NAD^+^: nicotinamide adenine dinucleotide (oxidised); NADH: nicotinamide adenine dinucleotide (reduced); NADP+: nicotinamide adenine dinucleotide phosphate (oxidised); NADPH: nicotinamide adenine dinucleotide phosphate (reduced); FAD: flavin adenine dinucleotide (oxidised); FADH_2_: flavin adenine dinucleotide (reduced); ACC: acetyl-CoA carboxylase; mTOR: mammalian target of rapamycin; SREBP1: sterol regulatory element-binding protein 1; SCD1: stearoyl-coenzyme A (CoA) desaturase-1; MUFAs: monounsaturated fatty acids; PUFAs: polyunsaturated fatty acids; FAs: fatty acids; NME1: nucleoside diphosphate kinase 1; NDP: nucleotide diphosphate; NTP: nucleotide triphosphate; PRIM2: DNA primase subunit 2; ETC: electron transport chain; e^−^: electron.

Canagliflozin also inhibits HCC cell proliferation through other mechanisms. Studies were conducted to assess the effect of canagliflozin on the growth and metabolic reprogramming of HCC using multi-omics analysis of metabolomics and absolute quantification proteomics (iMPAQT) [29]. Eight HCC cell lines were assessed, such as: Huh7, HLF, HepG2, Hep3B, KYN2, KMCH1, HAK1A and HAK1B. SGLT-2 expression was confirmed in all tested HCC cell lines. The expression of SGLT1 and GLUT1,2,3,5 and 6 was also confirmed in these cell lines. Canagliflozin, a specific SGLT-2 inhibitor, was shown to significantly inhibit the proliferation of Hep3B and Huh7 cells, although this was not because of a a reduction in intracellular glucose levels. Since glucose levels were not affected, it was assumed that this may be due to compensatory effects of other glucose transporters such as SGLT-1 and various GLUT types present in Hep3B and Huh7 cells. Thus, the inhibition of these cells by canagliflozin occurs in a mechanism independent of the inhibition of the SGLT-2 cellular glucose influx [29]. Multi-omics analysis show that canagliflozin causes, among other things, significant changes in oxidative phosphorylation and fatty acid metabolism. Canagliflozin indirectly activates adenosine monophosphate-activated protein kinase (AMPK) [108] (Figure 2). AMPK is a sensor of the ATP level [109,110,111,112,113]. Activation of AMPK occurs as a result of its phosphorylation at the Thr172 position within the activation loop in the α subunit. This phosphorylation reaction is catalysed by liver kinase B1 (LKB1) or calcium/calmodulin-dependent protein kinase kinase 2 (CAMKK2). CAMKK2 is activated by an increase in the concentration of calcium ions, while the activation of LKB1, and thus an increase in AMPK phosphorylation at the Thr172 position occurs as a result of an increase in AMP concentration. Canagliflozin, by inhibiting complex I in the mitochondrial electron transport chain and inhibiting oxidative phosphorylation reduces the production of ATP and thus increases the AMP/ATP ratio. This leads to an increase in Thr172 phosphorylation in the α subunit of AMPK and to its activation. Moreover, AMP can bind to the ɣ subunit of AMPK, thus directly activating AMPK through the allosteric mechanism [111,114,115,116].

Canagliflozin has been shown to affect oxidative phosphorylation by regulating various proteins associated with the electron transport system (Figure 2). Canagliflozin downregulates subunit α of ATP synthase F1 [29]. Additionally, in another study that assessed the anticancer activity of SGLT-2 inhibitors, it was shown that in concentrations of 5–30 µM canagliflozin, but not dapagliflozin, inhibits the proliferation and clonogenic survival of prostate (PC3, 22RV-1), lung (A549, H1299), liver (HepG2) and breast (MCF7) cancer cells [7]. In this study, it was shown that canagliflozin strongly and dose-dependently inhibits the mitochondrial complex-I without affecting complex-II. These same findings are confirmed by other researchers [7]. Inhibition of the mitochondrial complex-I has been shown to be essential for the anti-lipogenic and anti-proliferative effects of canagliflozin in treated cancer cells. It results in a disturbance of cellular respiration and a decrease in cellular ATP concentration and an increase in the AMP/ATP ratio, which, in turn, causes a rapid and significant increase in AMPK activity [7,111]. A fourfold increase in AMPK activity was observed within 30 min. The increase in AMPK activity, induced by canagliflozin, resulted in an eightfold increase in the phosphorylation of acetyl-CoA carboxylase (ACC) at Ser79 and, additionally, in the inhibition of mammalian target of rapamycin (mTOR). ACC is a regulator of fatty acid synthesis [29,111,117,118]. Phosphorylation of ACC leads to its inhibition and so, canagliflozin strongly suppresses fatty acid synthesis. ACC phosphorylation in HCC cells causes inhibition of both de novo lipogenesis and proliferation of these cells [119] (Figure 2). This inhibition of the synthesis of fatty acid derived from acetate, indicated that the inhibition of lipogenesis in the cancer cells studied was not dependent on the reduction of cellular glucose influx by canagliflozin. In the cell lines studied, the canagliflozin-induced increase in AMPK activity and in ACC phosphorylation was shown to be dose-dependent and associated with the inhibition of the proliferation and clonogenic survival of these cells. None of these effects were shown for dapagliflozin [7]. However, although the observed increase in AMPK activity under the influence of canagliflozin was significant and dose-dependent, blocking the ability of AMPK to phosphorylate and inhibiting ACC did not impair the inhibitory effect of canagliflozin on cancer cell proliferation. This suggests that AMPK activation-induced ACC phosphorylation is not critically important for the antiproliferative effect of canagliflozin. It was confirmed, however, that the inhibition of mitochondrial complex-I by canagliflozin is critical to this antiproliferative action. When the blockade of complex-I was bypassed by overexpression of reduced nicotinamide adenine dinucleotide (NADH) dehydrogenase, subunit 1, complex I (NDI1), canagliflozin did not inhibit the cancer cell proliferation. Nevertheless, since canagliflozin inhibits mitochondrial complex-I and reduces cellular glucose influx by inhibiting SGLT-2, SGLT-1 and GLUT-1, such a dual mechanism on cancer cell proliferation should be taken into consideration [7].

#### 3.2.2. Suppression of SREBP1 and Further Consequences

Canagliflozin Inhibits SREBP1 and SCD1 in Breast Cancer and HCC Cells through the Activation of AMPK.

SGLT-2 inhibitor-induced AMPK activation further results in sterol regulatory element binding protein 1 (SREBP1) being suppressed (Figure 2). SREBPs are a family of transcription factors which, through control of the expression of enzymes required for the synthesis of cholesterol, fatty acids, triacylglycerols and phospholipids, regulate lipid biosynthesis. The activation of AMPK inhibiting SREBP1 leads to the suppression of stearoyl-coenzyme A (CoA) desaturase 1 (SCD1) [120]. This results in the suppression of the production of monounsaturated fatty acids (MUFAs) and polyunsaturated fatty acids (PUFAs) become predominant which enhances lipid peroxidation and induces ferroptosis, which is regulated cell death. As a result of ferroptosis, the redox potential of the cell is disturbed which leads to oxidation of cellular lipids [121]. This damages the cell organelles and the cell membranes, increases cell entropy and, ultimately, leads to cell death. In recent years, oncologists have become particularly interested in ferroptosis as support for anticancer hormonal or drug therapy. Attempts are being undertaken to use mechanisms regulating ferroptosis to enhance the effectiveness of anticancer therapies. SCD1 expression has been shown to be present in human breast cancer samples and corresponds to poorer prognoses in patients with different types of cancer [122]. MUFAs inhibit ferroptosis by competitively interfering with the activity of PUFAs [123,124,125]. In contrast to MUFAs, PUFAs are preferential substrates for lipid peroxidation. Thus, the inhibition of SCD1, induced by SGLT-2 inhibitors, connects AMPK activation and SREBP1 inhibition to ferroptosis. SCD1 inhibition causes diminished production of MUFAs, resulting in a surplus of PUFAs and increased lipid peroxidation, which subsequently, causes ferroptosis [123] (Figure 2).

Canagliflozin also downregulates SCD1 in HCC cells, resulting in the inhibition of fatty acid synthesis and proliferation [29]. Downregulation of SCD1 is also known to inhibit prostate cancer cell proliferation by suppressing the production of MUFAs [126]. Another mechanism by which canagliflozin may exert its anticancer effects has also been confirmed in HCC cells. Canagliflozin significantly downregulates acetyl-coenzyme A acyl-transferase 1 (ACAT1), which is a key enzyme regulating fatty acid beta-oxidation and the formation of ketone bodies such as 3-hydroxybutyrate, among others [29]. ACAT1 is thought to promote the formation and progression of HCC [127,128]. Therefore, inhibition of ACAT1 by canagliflozin is one of its anticancer effect on HCC.

#### 3.2.3. Cell Cycle Arrest

Canagliflozin Leads to Cell Cycle Arrest in HCC Cells through the Activation of AMPK.

Canagliflozin-induced AMPK activation also leads to cell cycle arrest of Hep3B and HepG2 cells in the G2/M phase [29] (Figure 2). AMPK has been confirmed to induce G2/M arrest in liver cancer cells in a mechanism involving regulation at the levels of the transcription factor p53 and protein p21 [129,130].

#### 3.2.4. Other Effects of AMPK Activation

Canagliflozin and Dapagliflozin Inhibit mTOR in Breast and Pancreatic Cancer Cells through the Activation of AMPK.

As with the other cancer cell types described above, canagliflozin and dapagliflozin were also shown to strongly inhibit oxidative phosphorylation in breast cancer cells (MCF-7 and ZR-75–1), reduce ATP production, decrease intracellular ATP concentration and enhance AMPK phosphorylation at the Thr172 position. All these effects were dose-dependent and result in the inhibition of mTOR [47] (Figure 2). This suppression of mTOR is being investigated in various models as a potential treatment for breast cancer [131]. This signalling pathway leading through AMPK activation to mTOR inhibition is thought to be, at least partly, responsible for both suppression of the proliferation and inhibition of the cell cycle in G1 phase as well as induction of apoptosis in breast cancer cells [47]. It was confirmed that canagliflozin exerted its antiproliferative and apoptosis-inducing effects via the mTOR signalling pathway on pancreatic cancer cells as well [132]. Furthermore, the mechanism of the anticancer action of SGLT-2 inhibitors associated with AMPK activation was confirmed in cervical cancer cells [133]. In vitro studies using cervical cancer cell cultures showed that empagliflozin inhibits their migration and enhances apoptosis. At the same time, empagliflozin was confirmed to increase AMPK activity and to inhibit Sonic Hedgehog Signalling Molecule (SHH) expression in these cells representing the main mechanisms of empagliflozin’s anticancer action against cervical cancer [133].

### 3.3. Inhibition of DNA and RNA Synthesis

Canagliflozin Downregulates NME1 and Inhibits RNA and DNA Synthesis in HCC.

Studies using iMPAQT and multi-omics analysis of metabolomics have shown that canagliflozin downregulates nucleoside diphosphate kinase 1 (NME1) and upregulates nucleotide diphosphate (NDP) in Hep3B cells [29] (Figure 2). NME1 is the enzyme that catalyses the synthesis of nucleotide triphosphate (NTP) from NDP [134]. Thus, the suppression of NME1 by canagliflozin results in the upregulation of NDP in Hep3B. In a mouse model of HCC, NME1 and NME2 were shown to be upregulated [135]. It was confirmed that canagliflozin downregulates NME1 thereby inhibiting RNA and DNA synthesis [29]. Additionally, canagliflozin was shown to downregulate the expression of the DNA primase subunit 2 (PRIM2), interfering with DNA replication and mRNA transcription [29] (Figure 2). PRIM2 is a regulatory primase subunit, which is an enzyme involved in nucleotide formation and in DNA replication and transcription [136]. The question of whether the observed actions on HCC cells are specific to canagliflozin or whether other SGLT-2 inhibitors will also exhibit them remains unresolved. Dapagliflozin, tested in the same way, did not show an inhibitory effect on Hep3B cell proliferation [29]. There is also a lack of studies confirming this effect with canagliflozin on other HCC cell lines, with the exception of Hep3B.

### 3.4. Cell Cycle and Proangiogenic Activities

Canagliflozin Induces G2/M Arrest in HCC Cells and Inhibits Proangiogenic Factors in HCC.

**Figure 3:**
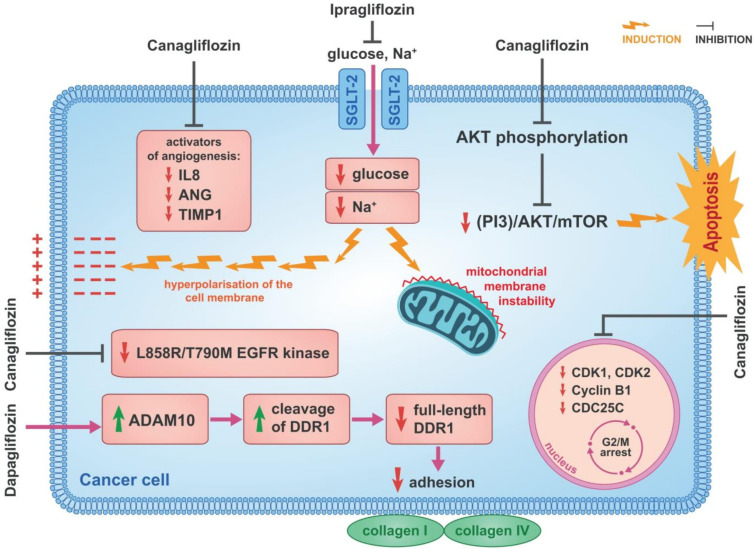
The mechanisms of anti-cancer action of SGLT-2 inhibitors. Canagliflozin induces G2/M arrest in Huh7 and HepG2 cells. In these cell types, the effects of canagliflozin on cycle-related genes were studied and the downregulation of genes such as CDK1, CDK2, Cyclin B1 and CDC25C was confirmed. Canagliflozin also inhibits AKT phosphorylation and by inhibiting the (PI3K)/AKT/mTOR signalling pathway induces apoptosis in Huh7 and HepG2 cells. Canagliflozin inhibits such activators of angiogenesis as IL-8, ANG and TIMP-1 in HCC cells which produce increased amounts of these activators. These activators of angiogenesis are involved in the growth and progression of HCC. It has been confirmed that ipragliflozin has an antiproliferative effect in breast cancer cells. Through the inhibition of cellular sodium influx, ipragliflozin induces both a hyperpolarisation of the cell membrane and mitochondrial membrane instability in breast cancer cells. It has been confirmed that canagliflozin inhibits L858R/T790M EGFR kinase and this is the mechanism of its anticancer activity in lung cancer cells. Dapagliflozin has been confirmed to reduce the amount of full-length DDR1 protein. It has also been shown to directly increase the activity of ADAM10, which induces the cleavage of DDR1 in HCT116 cells. In this way dapagliflozin disrupts the adhesive abilities of HCT116 cells to collagen I and IV. Explanation of abbreviations: SGLT-2: sodium-glucose cotransporter 2; AKT: protein kinase B; PI3K: phosphatidylinositol 3-kinase; mTOR: mammalian target of rapamycin; IL-8: interleukin-8; ANG: angiogenin; TIMP-1: tissue inhibitors of metalloproteinase-1; EGFR: epidermal growth factor tyrosine; ADAM10: a disintegrin and metalloproteinase domain-containing protein 10; DDR1: discoidin domain receptor family, member 1.

Canagliflozin has an antiproliferative effect on HCC cells. Kosuke Kaji and his colleagues confirmed, in cultures of Huh7 and HepG2 cells, that canagliflozin inhibits proliferation of these cells and that effect is dose-dependent [6]. This was not seen in HLE cells. In the same study, canagliflozin was shown to inhibit cellular glucose transport in Huh7 and HepG2 cells, but not in SGLT-2-non-expressing HLE cells nor in primary human hepatocytes (PHH). Moreover, canagliflozin was confirmed to significantly reduce intracellular ATP levels in Huh7 and HepG2 cells. The authors of this study emphasise that the antiproliferative effect of canagliflozin on HCC cells is mediated by mechanisms dependent on cellular glucose influx by SGLT-2 [6]. This is in line with previous studies on human melanoma cells, which showed pharmacological inhibition of cellular glucose influx results in the cell cycle arrest in the G2/M phase [137]. Canagliflozin induces G2/M arrest in Huh7 and HepG2 cells (Figure 3). In these cells, the effects of canagliflozin on cycle-related genes were studied and downregulation of CDK1, CDK2, Cyclin B1 and CDC25C as well as the upregulation of checkpoint kinase 1 (CHK1) and p21 were confirmed [6]. The ability of canagliflozin to induce cell cycle arrest is further indicated by its inhibitory effects on extracellular signal-regulated kinase (ERK) 1/2, p38 and protein kinase B (AKT) phosphorylation. The ability of canagliflozin to induce apoptosis in Huh7 and HepG2 cells via caspase3 activation was also demonstrated [6]. AKT is a regulator of glycolysis and plays an important role in cancer cell survival. It has been shown that activated AKT can stimulate cellular glucose influx and aerobic glycolysis in human glioblastoma cells [138]. The importance of AKT has been further underlined by a study showing that the phosphatidylinositol 3-kinase (PI3K)/AKT/mTOR signalling pathway regulates apoptosis in a caspase3-dependent mechanism and further stabilises hypoxia inducible factor 1 subunit alpha (HIF-1α), which is a known regulator of genes involved in the transport and metabolism of glucose [139]. This indicates that canagliflozin’s inhibition of AKT phosphorylation may be an important mechanism for its apoptosis-inducing effects in Huh7 and HepG2 cells (Figure 3).

In a mouse model of a human liver cancer xenograft, canagliflozin was shown to inhibit neovascularisation in both Huh7- and HepG2-derived tumours. This seems particularly relevant, in view of the strong link between glucose metabolism and tumour angiogenesis which has been confirmed [140,141,142]. Tumour cells produce and secrete angiogenesis activators to stimulate angiogenesis to ensure their oxygen and nutrient supply [143]. These proangiogenic factors include basic fibroblast growth factor (bFGF) and vascular endothelial growth factor (VEGF) and are upregulated by glycolysis end products such as lactate and pyruvate [144,145,146] which are similar to the production profile of angiogenesis-related cytokines and chemokines produced in cultures of Huh7 and HepG2 cells. Increased production of such activators of angiogenesis as interleukin-8 (IL-8), angiogenin (ANG) and tissue inhibitors of metalloproteinase-1 (TIMP-1) was found and, at the same time, inhibition by canagliflozin was confirmed [6] (Figure 3). The involvement of proangiogenic factors in the growth and progression of HCC was shown by others [147,148,149,150,151,152]. Ten weeks after administration of canagliflozin in a patient with cirrhosis and diabetes, a spontaneous regression of HCC was reported, which was linked, in this patient, to a significant downregulation of some angiogenesis-related cytokines [153]. Under the influence of canagliflozin, downregulation of matrix metalloproteinase-8 (MMP-8), angioprotein-1, angioprotein-2, prolactin and placenta growth factor-AA (PGF-AA) occurred. Spontaneous tumour regression is a rare and poorly understood phenomenon and the described case may indicate that SGLT-2 inhibitors can cause HCC regression by inhibiting angiogenesis [153].

### 3.5. Inhibition of Cellular Sodium Influx

Ipragliflozin Inhibits Cellular Sodium Influx in Breast Cancer Cells.

The antiproliferative effects of both canagliflozin and dapagliflozin have been confirmed in breast cancer [12,47]. In studies using MCF-7 and ZR-75-1 cell cultures, canagliflozin and dapagliflozin were shown to dose-dependently inhibit proliferation and growth of these cells. In addition, tests with SGLT-2 inhibitors inhibited clonogenic survival of MCF-7 cells [47]. This study confirmed a reduction in cellular glucose influx in MCF-7 and ZR-75-1 breast cancer cells, indicating involvement of SGLT-2 inhibition in the anticancer effects of canagliflozin and dapagliflozin on breast cancer. This seems to be significant because, as shown in this study, the expression of SGLT-2 in breast cancer cells is considerable. This was confirmed in a study with ipragliflozin, the SGLT-2 inhibitor used in Japan, which confirmed the antiproliferative effect in the breast cancer cell line MCF-7 [1]. This effect was dose-dependent and eliminated by knocking down SGLT-2 expression using siRNA. They further pointed out that this suppression was not only due to cellular glucose influx but also to the accompanying cellular sodium influx. The importance of cellular sodium influx for breast cancer growth has been recently highlighted [154]. For this reason, exploiting the mechanism of action of SGLT-2 inhibitors is of particular interest. They showed that ipragliflozin, by inhibiting cellular sodium influx, induces both a hyperpolarisation of the cell membrane and mitochondrial membrane instability in MCF-7 cells (Figure 3). These effects underline the importance of this antiproliferative mechanism of canagliflozin on breast cancer cells [1].

### 3.6. Disruption of Glutamine Metabolism

Canagliflozin Significantly Inhibits GDH activity in Breast Cancer Cells.

The antiproliferative effects of canagliflozin and dapagliflozin were also tested on breast cancer cell lines that have high levels of cellular glucose uptake and a high degree of aerobic glycolysis [12]. These were two human breast cancer lines, SKBR3 and BT-474, which were chosen because of their expected high degree of sensitivity to drugs that inhibit cellular glucose influx. Canagliflozin and, to a lesser extent, also dapagliflozin have been shown to inhibit the proliferation of these cells, and also did that in a glucose-free environment. The potency of the antiproliferative effect of canagliflozin and dapagliflozin was similar in both presence and absence of glucose. This suggests that the antiproliferative effect of these drugs is independent of the level of cellular influx of glucose and points to other mechanisms of antiproliferative SGLT-2 inhibitor action independent of SGLT-2 inhibition [12]. In studies using breast cancer cells, canagliflozin was shown to interfere with cellular respiration and ATP production by disrupting glutamine utilisation [12]. Glutamine is converted to glutamate, which is the carbon source for the tricarboxylic acid (TCA) cycle. The enzyme that shuttles glutamate into TCA is glutamate dehydrogenase (GDH), which converts glutamate into α-ketoglutarate. High GDH activity enhances breast cancer proliferation and is considered a marker of poor prognosis in this type of cancer [155]. GDH activity is associated with the ability of breast cancer cells to adapt to metabolic stress [156,157]. It regulates redox homeostasis and thus promotes the proliferation of these cells [12]. In contrast, decreasing GDH activity significantly inhibits breast cancer cell proliferation. It has been shown that canagliflozin, which increases the cellular influx of glutamine, significantly inhibits GDH activity, resulting in an increase in intracellular glutamate and a decrease in intracellular α-ketoglutarate concentration [12]. Hence, the decrease in α-ketoglutarate concentration results in a reduction in its incorporation into TCA and ATP production with further consequences (Figure 2). Canagliflozin’s inhibition of GDH is an important antiproliferative mechanism and this is further emphasised by the fact that this is partially inhibited by supplementation with dimethyl-oxoglutarate, which bypasses the blockade at the level of GDH and restores the correct level of α-glutarate in cells. Thus, disruption of glutamine metabolism is an important mechanism for the antiproliferative action of canagliflozin in breast cancer cells [12].

### 3.7. Inhibition of EGFR

Canagliflozin Inhibits L858R/T790M EGFR Kinase in Lung Cancer Cells.

In non-small cell lung cancer (NSCLC), the ability of canagliflozin to induce apoptosis by a mechanism independent of SGLT-2 inhibition and cellular glucose influx was demonstrated [158]. Some studies were performed on H1975 cells which harbour an epidermal growth factor tyrosine (EGFR) L858R/T790M mutation. This mutation causes resistance to epidermal growth factor tyrosine kinase inhibitors (EGFR TKIs) which are commonly used in the treatment of advanced lung cancer. In this study it was shown by significantly impairing the effectiveness of treatment with EGFR TKIs, canagliflozin inhibits L858R/T790M EGFR kinase anticancer activity in lung cancer cells that are resistant to EGFR TKIs [158] (Figure 3).

### 3.8. Reduction of Cancer Cell Adherence

Dapagliflozin Disrupts Adhesion of Human Colon Carcinoma Cells.

SGLT-2 inhibitors also interfere with the adhesion capacity of different cancer cell types [88]. The effects of dapagliflozin, empagliflozin and tofogliflozin were evaluated in human colon carcinoma (HCT116), human hepatocyte carcinoma (HepG2), pancreatic cancer (PANC-1) and lung cancer (H1792) cells. Of the SGLT-2 inhibitors tested, only dapagliflozin caused loss of cancer cell adhesion which was attributed to altered metabolism and inactivation of dapagliflozin. Its unique property compared to other SGLT-2 inhibitors is that it binds to glucuronic acid via UDP-glucuronyltransferase Family 1 Member A9 (UGT1A9), which causes both dapagliflozin inactivation and excretion. In contrast, empagliflozin is inactivated and excreted not only by UGT1A9 but also by UDP-glucuronylotransferase Family 2 Member B7 (UGT2B7), UDP-glucuronylotransferase Family 1 Member A3 (UGT1A3) and UDP-glucuronylotransferase Family 1 Member A8 (UGT1A8). Tofogliflozin, in turn, is inactivated and excreted by various cytochrome P-450-related enzymes, such as cytochrome P-450 family 2 subfamily C member 18 (CYP2C18) and cytochrome P-450 family 3 subfamily A member 4 (CYP3A4), among others. As the adhesion-blocking effect of dapagliflozin on cancer cells was only demonstrated in HCT116 cells while other cancer cell types tested were resistant to such effects, the expression of SGLT-2 and UGT1A9 was examined in all these cell types. HCT116 cells expressed SGLT-2 approximately 5 times higher than H1792 cells and approximately 10 times higher than HepG2 and PANC-1 cells. On the other hand, UGT1A9 expression was found to be approximately 20 times lower in HCT116 cells than in HepG2 cells. A low expression of UGT1A9 was also confirmed in PANC-1 and H1792 cells [88]. The results of this study indicated that the anti-adhesion effect of dapagliflozin in relation to cancer cells depends on the expression levels of SGLT-2 and UGT1A9. Panc-1 and H1792 cells, which have low SGLT-2 and UGT1A9 expression, were resistant to the anti-adhesive effect of dapagliflozin. In contrast, HCT116 cells, having the highest level of SGLT-2 and the lowest level of UGT1A9 expression, were most sensitive to the anti-adhesive effect of dapagliflozin. It is thought that this high ratio of SGLT-2 to UGT1A9 expression in these cells allows dapagliflozin to have an anti-adhesive effect because it is not inactivated by UGT1A9 and can act through SGLT-2. This effect was dose-dependent. Conversely, increased expression of UGT1A9 in these cells resulted in decreased, dapagliflozin-induced cell detachment [88]. When the mechanisms of this anti-adhesive effect of dapagliflozin in comparison with HCT116 cells were investigated in more detail, it was shown that dapagliflozin selectively interferes with the adhesion to collagen I and IV, but not to fibronectin, vitronectin and laminin. Collagen I and IV bind to discoidin domain receptor family, member 1 (DDR1), which activates their intrinsic tyrosine kinase activity. Dapagliflozin was confirmed to reduce the amount of full-length DDR1 protein by increasing the activity of a disintegrin and metalloproteinase domain-containing protein 10 (ADAM10), which induces the cleavage of DDR1 [88] (Figure 3). The mechanisms of the anticancer action of SGLT-2 inhibitors are summarised in Table 2.

## 4. SGLT-2 Inhibitors and Other Anticancer Drugs

The aforementioned AMPK-mTOR signalling pathway is also a central mechanism regulating intracellular autophagy or intracellular bulk degradation system, which is regarded as the mechanism that determines cell survival while its disruption is associated with non-apoptotic cell death [159,160,161]. Sunitinib, a multi-targeted tyrosine inhibitor, is an anticancer drug commonly used to treat tumours such as renal cell carcinomas and gastrointestinal stromal tumours [162,163]. Its major side effect is cardiotoxicity and heart failure [164,165]. Sunitinib has been shown to significantly interfere with autophagy in cardiomyocytes by inhibiting AMPK phosphorylation [166,167,168]. Both in vitro studies in H9c2 cardiomyocyte cultures and in vivo studies in a mouse model of sunitinib-induced left ventricular systolic dysfunction confirmed that sunitinib significantly inhibits AMPK phosphorylation and activates mTOR. This results in the inhibition of late-stage autophagy in cardiomyocytes and is the primary mechanism of sunitinib’s cardiotoxic effects [166]. These actions of sunitinib were eradicated by empagliflozin, which restored AMPK activation and inhibited mTOR. Thus, empagliflozin restored sunitinib-disrupted autophagy flux in cardiomyocytes and reversed the cardiotoxic effects [166]. The strong protective effect of empagliflozin on cardiomyocytes demonstrated in these studies may have important clinical implications. However, this requires further in vivo studies. Whether the use of empagliflozin together with sunitinib in cancer treatment will affect the anticancer efficacy of sunitinib also remains to be clarified [166].

**Figure 4:**
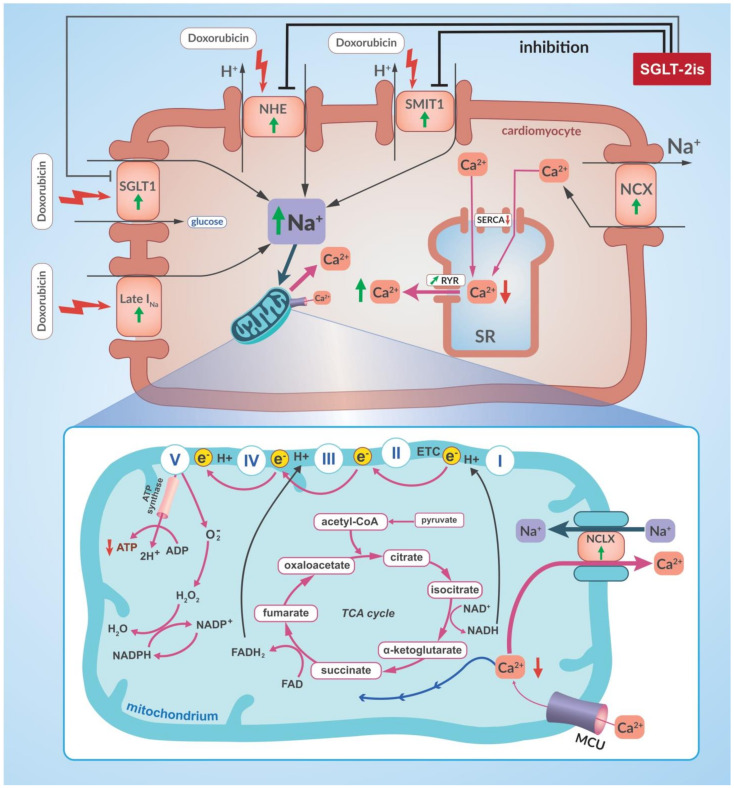
SGLT-2 inhibitors inhibit the cardiotoxic action of doxorubicin. SGLT-2 inhibitors reduce the overload of cardiomyocytes with calcium ions which occurs as a result of doxorubicin and which is one of the known mechanisms of the cardiotoxic effects of doxorubicin. This doxorubicin-induced calcium ion overload of cardiomyocytes, as in heart failure, is closely related to their sodium ion overload, and this in turn results from an overexpression of SGLT-1, increased Na^+^ influx by late I_Na_ and increased NHE and SMIT1 activity. This overloading of the cytoplasm of the cardiomyocytes with sodium causes, with the involvement of NCLX, an excessive efflux of calcium ions from the mitochondria. At the same time, there is an impaired influx of calcium ions into the sarcoplasmic reticulum involving SERCA, whose activity is reduced, and an excessive efflux of calcium ions from the sarcoplasmic reticulum involving RYR. This leads to an excessive overload of cardiomyocyte cytoplasm by calcium ions and to a fall in the calcium ion concentration inside the mitochondria. This decrease in calcium ion concentration inside the mitochondria results in dysregulation of the activity of the calcium ion-dependent dehydrogenases associated with the TCA cycle. This results in impaired production of NADH and NADPH. Decreased NADH concentration causes a decrease in ATP production in the mitochondrial electron transfer chain, while decreased NADPH concentration causes a less efficient mitochondrial antioxidant defence. In addition to their partial affinity for SGLT-1, SGLT-2 inhibitors also can also directly inhibit NHE and SMIT1 in cardiomyocytes and therefore can rebalance the intracellular concentration of sodium ions and calcium ions in cardiomyocytes by this very mechanism. Explanation of abbreviations: SGLT-2is: sodium-glucose cotransporter-2 inhibitors; SR: sarcoplasmic reticulum; SERCA: sarco/endoplasmic reticulum Ca^2+^—ATPase; RYR: ryanodine receptors; SGLT1: sodium-glucose cotransporter-1; NHE: sarcolemmal Na^+^/H^+^ exchanger; SMIT1: sodium—myoinositol cotransporter-1; Late I_Na_: late Na^+^ current; NCX: sarcolemmal Na^+^/Ca^2+^ exchanger; NCLX: mitochondrial Na^+^/Ca^2+^ exchanger; MCU: mitochondrial Ca^2+^ uniporter; ATP: adenosine triphosphate; ADP: adenosine diphosphate; TCA cycle: tricarboxylic acid cycle; NAD^+^: nicotinamide adenine dinucleotide (oxidised); NADH: nicotinamide adenine dinucleotide (reduced); NADP^+^: nicotinamide adenine dinucleotide phosphate (oxidised); NADPH: nicotinamide adenine dinucleotide phosphate (reduced); FAD: flavin adenine dinucleotide (oxidised); FADH_2_: flavin adenine dinucleotide (reduced); ETC: electron transport chain; e^−^: electron.

The cardiomyocyte-protective effect of empagliflozin has also been shown regarding the cardiotoxic effects of doxorubicin [169]. In a study using non-diabetic mice in which myocardial dysfunction was induced with doxorubicin, it was shown that the concomitant use of empagliflozin significantly reduced the extent of the myocardial damage [169]. In various ways empagliflozin was shown to attenuate the cardiotoxic effects of doxorubicin. The hearts of the studied mice showed an empagliflozin-induced decrease in mitochondrial lipid peroxidation markers, cytosolic malondialdehyde (MDA) concentration and in the expression of pro-inflammatory cytokines such as inteleukin-1 β (IL-1 β), interleukin-6 (IL-6) and interleukin-8 (IL-8). Moreover, markers of myocardial fibrosis such as collagen and MMP-9 expression and markers of cardiomyocyte apoptosis, such as the number of apoptotic nuclei and caspase-3 expression induced by doxorubicin, were considerably reduced when empagliflozin was administered [169]. Mouse HL-1 cardiomyocytes, oestrogen-responsive breast cancer cell line MCF-7 and the triple-negative breast cancer (TNBC) cell line MDA-MB-231 were evaluated in cell culture [169]. Under doxorubicin treatment a significant reduction of HL-1 celll viability was observed and this effect was dose-dependent. Simultaneous exposure of cardiomyocytes to doxorubicin and empagliflozin was associated with a significantly higher viability of cardiomyocytes than when exposed to doxorubicin alone and this protective effect of empagliflozin was also dose-dependent [169]. It was shown that empagliflozin reduces calcium ion overload in cardiomyocytes, which is one of the cardiotoxic effects of doxorubicin [170,171]. Doxorubicin-induced calcium ion overload of cardiomyocytes, as in heart failure, is closely related t sodium ion overload, which is due to an overexpression of SGLT-1, increased Na^+^ influx by the sodium chanel late carrent (Late I_Na_) and increased activity of the sarcolemmal Na^+^/H^+^ exchanger (NHE) and sodium-myoinositol cotransporter-1 (SMIT-1) [28] (Figure 4). SGLT-2 inhibitors such as empagliflozin, canagliflozin and dapagliflozin, in addition to their partial affinity for SGLT-1, have the ability to directly inhibit NHE and SMIT1 in cardiomyocytes [27,28,172,173,174]. SGLT-2 inhibitors are, therefore, able to rebalance intracellular sodium ion and calcium ion concentrations in cardiomyocytes (Figure 4). This is also supported by results from a study using a rat model of myocardial infarction, where empagliflozin was shown to restore calcium ion homeostasis in cardiomyocytes through this mechanism and improve left ventricular systolic function after myocardial infarction [173]. This confirms that empagliflozin can restore the correct level of calcium ions in cardiomyocytes in both myocardial infarction—induced heart failure and doxorubicin-induced cardiomyocyte calcium overload.

However, it was shown in breast cancer cells, that empagliflozin does not reduce the anticancer effect of doxorubicin but, at higher concentrations, it even increases the cytotoxic effect of doxorubicin [169]. Similar results were obtained in studies using the triple-negative breast cancer cell line MDA-MB-231. which are characterised by an aggressive phenotype, high metastatic capacity and a poor prognosis [175]. An additional problem is the frequent occurrence of secondary multi-drug resistance (MDR) in TNBC, which includes resistance to doxorubicin. The mechanism of MDR is thought to be an increased expression of the ATP-dependent efflux pump ABCB1 (MDR1). In vitro, empagliflozin did not interfere with the cytotoxic effects of doxorubicin on MDA-MB-231 cells, and, furthermore, a combination of empagliflozin and doxorubicin resulted in decreased MDR1 expression and a subsequent increased sensitivity to doxorubicin [175]. The molecular mechanisms of this empagliflozin action have been shown to include mainly a decrease in mTOR and BCL gene expression and an upregulation of pro-apoptotic gene expression (p21). The mechanism of action of empagliflozin, blocking the receptor for calmodulin, is also noteworthy [175]. Together, these three mechanisms mentioned above contribute to an increased sensitivity of breast cancer cells to doxorubicin, suggesting that the combined use of empagliflozin with doxorubicin will permit a lower dosage of doxorubicin thus reducing its toxic side effects and especially its cardiotoxicity [175].

Canagliflozin also increases the sensitivity of cancer cells to the cytotoxic effects of doxorubicin [176]. This was shown in vitro on HepG2 and HepG2-ADR (adriamycin or doxorubicin—resistant) HCC and, also in MCF7 breast cancer cells. Canagliflozin significantly reduced levels and inhibited the P-glycoprotein (P-gp) function in HepG2 cells, which contributed to an increased uptake as well as an increased doxorubicin concentration in these cells. P-gp function is critically dependent on ATP levels. Canagliflozin significantly reduces intracellular ATP levels and thus interferes with P-gp function, contributing to cytotoxic effects of doxorubicin on cancer cells [176].

There are also reports describing the use of SGLT-2 inhibitors for the treatment of severe hyperglycaemia, the most common adverse effect of using alpelisib, [177,178]. The use of SGLT-2 inhibitor, dapagliflozin was described for single cases, where there was very good glycaemic control which permitted the continuation of effective anti-tumour therapy with alpelisib [177]. Thus, SGLT-2 inhibitors may prove to be helpful in advanced breast cancer. Clearly, further studies are needed to confirm the efficacy and safety of such treatments. There is also a need to identify risk factors for euglycemic ketoacidosis, a rare complication reported in isolated cases of canagliflozin-treated diabetes which was induced by another PI3K inhibitor, taselisib [179].

## 5. Clinical Importance and Perspectives

In view of the importance of metabolic reprogramming of cancer cells for tumour progression and their resistance to treatment, researchers are focusing on different elements of this reprogramming, viewing them as potential therapeutic targets. Therefore, the possibility of blocking glucose uptake by cancer cells using SGLT-2 inhibitors appears to be an attractive therapeutic approach, especially in view of the expression of SGLT-2 confirmed in numerous types of cancer cells. Furthermore, SGLT-2 inhibitors are now registered and widely used in the treatment of diabetes and heart failure. Knowing the enhanced glucose dependency of tunours (Warburg effect), the interest of researchers in SGLT-2 inhibitors as a group of drugs with anticancer activity has risen in recent years. Besides blocking SGLT-2 various mechanisms of anticancer action of this group of drugs are being investigated. As outlined above, these mechanisms are diverse and often independent of the inhibition of cellular glucose influx by SGLT-2. Some of them appear to be particularly important as they target important cancer-driving mechanisms and may enable new ways of treating certain cancers in the future. One such mechanism of action of SGLT-2 inhibitors is, for example, their inhibitory effect on intracellular ATP production, which results in a considerable activation of AMPK in cancer cells. This, in turn, has multidirectional effects (Figure 2). Recently, research has been carried out attempting to utilise the mechanisms regulating ferroptosis in order to improve the efficacy of anticancer therapies [122]. SGLT-2 inhibitor-induced AMPK activation leads to inhibition of mTOR and induction of apoptosis [7,47]. The suppression of mTOR is being explored in various models as a possible treatment for breast cancer [131]. It is believed that this signalling pathway, is, at least, partially responsible for the effects of canagliflozin and dapagliflozin on both suppressing the proliferation and arresting the cell cycle in the G1 phase, as well as for inducing apoptosis in breast cancer cells [47]. Canagliflozin showed these antiproliferative and apoptosis-inducing effects via the mTOR signalling pathway in pancreatic cancer cells [132].

Another important mechanism of SGLT-2 inhibitor action is the aforementioned effect of canagliflozin, on the β-catenin signalling pathway in HCC cells, which represents a potential therapeutic target (Figure 1). Over-activation of the Wnt/β-catenin signalling pathway is a predisposing factor for HCC development [89,105].

It also seems worth exploiting the cardioprotective effects of SGLT-2 inhibitors, which may reduce the cardiotoxic effects of some anticancer drugs. This has been confirmed, for example, for empagliflozin used in combination with sunitinib whose mechanisms of cardiotoxic action are described above [162,163,166]. This does, however, require further research. Importantly, clarification is required as to whether prescribing empagliflozin together with sunitinib will weaken the potency of the anticancer effect of sunitinib.

This protective effect of empagliflozin on cardiomyocytes has also been demonstrated for the cardiotoxic effects of doxorubicin [169]. The mechanisms of empagliflozin’s protective effect in relation to cardiomyocytes are described above and shown in Figure 4. Most importantly, the potency of the anticancer effect of doxorubicin in breast cancer cells was not reduced by empagliflozin. In fact, the use of empagliflozin together with doxorubicin increases the sensitivity of breast cancer cells to doxorubicin [175]. This shows that empagliflozin together with doxorubicin may enable an improvement in the efficacy of doxorubicin treatment in the future and that lower doses of doxorubicin can be used, thus minimising its cardiotoxic effects.

New technologies are also currently being developed to enable more precise delivery of SGLT-2 inhibitors to the tumour and thus increase the effectiveness of anticancer therapy. Previously, special magnetically guided dapagliflozin nanoparticles were developed that, in combination with chemotherapy and radiotherapy, were used to treat therapy-resistant tumours [180]. Recently, a new generation of magnetic iron oxide nanoparticles coated with poly(methacrylic acid)-graft-poly(ethyleneglycol methacrylate) copolymer (IO/PMAA-g-PEGMA) have been developed in which canagliflozin was covalently coupled to the PMAA-g-PEGMA polymer. This yields CANA-loaded IO/PMAA-g-PEGMA nanoparticles with an average diameter of 68 nm, with very good physicochemical properties, colloidal stability and good drug release parameters [181]. The anticancer efficacy of CANA-loaded IO/PMAA-g-PEGMA nanoparticles was evaluated both in vitro in human lung cancer cell lines A549 (ATCC) and mus musculus (mouse) epidermal cancer cell lines PDVC57 and in vivo in a mouse xenograft cancer model. It was shown that CANA-loaded IO/PMAA-g-PEGMA nanoparticles exhibited a higher cytotoxicity towards A549 and PDVC57 cells than free canagliflozin and this was associated with a higher degree of apoptosis induced by the CANA-loaded IO/PMAA-g-PEGMA nanoparticles. The highest efficacy, in terms of tumour growth inhibition, was obtained when CANA-loaded IO/PMAA-g-PEGMA was combined with radiotherapy with the simultaneous application of an external magnetic field to the tumour area [181]. This requires further research to determine the optimal doses of both canagliflozin and radiation, but there is the prospect of using SGLT-2 inhibitors in combination with radiotherapy and, with this type of technology, to treat solid hypoxic tumours, which often develop resistance to chemo- and radiotherapy.

The abovementioned mechanisms may be of the greatest significance for future clinical application of these drugs. Although these mechanisms appear to be attractive therapeutic targets and raise hopes for their use in the treatment of various types of cancer, many important issues remain unexplained and further research is needed to clarify them. Such an issue is, for example, the dosage of individual SGLT-2 inhibitors if used in patients with oncological indications. Essentially, the effects of SGLT-2 inhibitors, which include the inhibition of cancer cell proliferation, have been demonstrated in in vitro studies, which have often used higher concentrations of these drugs than the maximum blood serum concentrations in patients using SGLT-2 inhibitors for diabetes or heart failure. Much higher doses of SGLT-2 inhibitors would be needed to achieve the same serum concentrations in patients that were used in some in vitro studies. Such doses have not been used in clinical trials in patients with diabetes or heart failure, and thus the short- and long-term safety of such doses of SGLT-2 inhibitors is unknown. Therefore, future preclinical and clinical studies to determine the safety and efficacy of anticancer therapy based on the use of SGLT-2 inhibitors are necessary.

## 6. Conclusions

Despite considerable progress in the treatment of cancer, it is the second leading cause of death globally, accounting for an estimated 9.6 million deaths, or one in six deaths, in 2018 [182]. Therefore, new and more effective ways of treatment are constantly being sought, especially since some types of cancer are characterised by extremely aggressive growth, metastasis and resistance to treatment. Researchers are interested in the possibility of using selective SGLT-2 inhibitors to block glucose uptake by cancer cells. The mechanisms of the anticancer action of this group of drugs are complex and still not fully understood. However, the possibility of employing drugs, which are already currently used in the treatment of diabetes and heart failure, for cancer treatment appears to be very attractive.

The use of SGLT-2 inhibitors may prove to be very beneficial in terms of improving the safety of previously used anticancer therapies. This applies to the cardioprotective effect of SGLT-2 inhibitors, particularly empagliflozin, during treatment with sunitinib and doxorubicin. Further studies are needed to confirm this effect in humans and determine the optimal way to use SGLT-2 inhibitors together with doxorubicin to maximally eliminate its cardiotoxic effect. Studies have shown that SGLT-2 inhibitors increase sensitivity of breast cancer cells to doxorubicin [175]. This gives rise to the assumption that the combined use of empagliflozin with doxorubicin will permit the use of lower doses of doxorubicin and will reduce its cardiotoxic effects. Furthermore, the synergistic effect of SGLT-2 inhibitors and doxorubicin, as demonstrated in breast cancer cells, makes it conceivable that this synergism with SGLT-2 inhibitors will also be confirmed in other tumour types and with other classic anticancer drugs. However, preclinical and clinical studies are needed to assess the possibility of using the synergistic effects of these drugs in clinical oncology in the future.

In addition, the new technologies described above, which allow selective accumulation of canagliflozin within the tumour and thus increase the effectiveness of the anticancer action of canagliflozin, especially in combination with radiotherapy, is a significant step forward on the way towards the effective and safe use of SGLT-2 inhibitors in oncology. The cited studies showing the synergistic effect of canagliflozin and radiotherapy open the way for further research to develop this technology and optimise the dosage of both canagliflozin and radiation [181]. Further research is also required to identify the tumour types that respond best to such treatments and to assess the feasibility of using this method to deliver other SGLT-2 inhibitors and perhaps other anticancer drugs. This offers the prospect of improving treatment efficacy especially for those cancers that develop resistance to chemo- and/or radiotherapy.

As diabetic patients have a higher risk of cancer, the question also arises whether diabetic patients who are diagnosed with cancer should have their diabetes therapy modified to include SGLT-2 inhibitors. Given the potential anticancer effect of SGLT-2 inhibitors, such treatment could be of clinical benefit to these patients. However, clinical trials are needed to assess the clinical benefits of doing this.

As can be seen, there are many areas and potential targets for the anticancer action of SGLT-2 inhibitors. However, many questions remain unresolved, mainly regarding the safety and clinical feasibility of using SGLT-2 inhibitors in cancer treatment. Nonetheless, numerous ongoing preclinical studies and probable new clinical trials to come give hope for the future optimal use of this interesting group of drugs in clinical oncology.

## Figures and Tables

**Table 1:** Summary of clinical trials assessing the cancer risk associated with SGLT-2 inhibitor therapy.

First Author, Publication Year, Country	Study Design	Cancer Type	No. of Participants	Type of SGLT-2 Inhibitors	Comparator	Reported OR or IRR or HR or RR (95% CI)	Notes
Ptaszyńska, 2015, Poland [81]	Case study	All cancer types	9339	Dapagliflozin	Placebo or other active treatment	IRR 1.035 (0.724, 1.481), *p* = 0.849	21 phase 2b/3 clinical trials, duration 12–208 weeks
Tang, 2017, China [5]	Pairwaise meta-analysis	All cancer types	34,569	Canagliflozin Dapagliflozin Empagliflozin	Placebo or other active treatment	OR 1.14 (0.96, 1.36), *p* = 0.60	46 indenedent randomized controlled trials, mean duration 61 weeks
Dicembrini, 2019, Italy [76]	Meta-analysis	All cancer types	48,185	Canagliflozin Dapagliflozin Empagliflozin Ertugliflozin	Placebo or other active treatment	OR 0.98 (0.77, 1.24), *p* = 0.82	27 randomized controlled trials with duration at least 52 weeks, mean duration 84 weeks
Suissa, 2021, Canada [71]	Primary analysis	Breast cancer	46,569	Canagliflozin Dapagliflozin Empagliflozin	DPP-4 inhibitors	HR (95% CI) 1.0 (0.76, 1.30)	Median follow-up 2.6 years
Shi, 2021, China [60]	Meta-analysis	All cancer types	88,973	Canagliflozin Dapagliflozin Empagliflozin Ertugliflozin Tofogliflozin Bexagliflozin	Placebo or other active treatment	RR (95% CI) 1.05 (0.97, 1.14), *p* = 0.20	77 randomized controlled trials with duration from 10 to 416 weeks
Dąbrowski, 2021, Poland [59]	Meta-analysis	All cancer types	66,568	Canagliflozin Dapagliflozin Empagliflozin Ertugliflozin Sotagliflozin	Placebo	RR (95% CI) 1.11 (0.98, 1.26), *p* = 0.10	8 cardiovascular and renal randomized controlled trials
Benedetti, 2021, Italy [87]	Meta-analysis	All cancer types	48,985	Canagliflozin Dapagliflozin Empagliflozin Ertugliflozin	Placebo	RR (95% CI) 0.35 (0.33, 0.37), *p* = 0.00	20 randomized clinical trials, with a particularly reduced risk of cancer for dapagliflozin and ertugliflozin (RR 0.06, CI 0.06–0.07 and RR 0.22, CI 0.18–0.26, respectively)

**Table 2:** Mechanisms of anticancer action of SGLT-2 inhibitors.

Cancer Type	Type of SGLT-2 Inhibitor Drug	Mechanism of Action
Hepatocellular carcinoma (HCC)	Canagliflozin	Inhibition of the action of β-catenin
HCC, prostate cancer, lung cancer, liver cancer and breast cancer	Canagliflozin	Inhibition of complex I and subunit α of ATP synthase F1 in the mitochondrial electron transport chain
Breast cancer, HCC	Canagliflozin	Suppression of SREBP1 and SCD1
HCC	Canagliflozin	Cell cycle arrest
Breast cancer, pancreatic cancer	Canagliflozin	Inhibition of mTOR
HCC	Canagliflozin	Inhibition of DNA and RNA synthesis
HCC	Canagliflozin	Induction of G2/M arrest and inhibition of proangiogenic factors
Breast cancer	Ipragliflozin	Inhibition of cellular sodium influx
Breast cancer	Canagliflozin	Disruption of glutamine metabolism
Lung cancer	Canagliflozin	Inhibition of L858R/T790M EGFR kinase
Human colon carcinoma	Dapagliflozin	Reduction in the adhesion of cancer cells

## References

[1] Komatsu S., Nomiyama T., Numata T., Kawanami T., Hamaguchi Y., Iwaya C., Horikawa T., Fujimura-Tanaka Y., Hamanoue N., Motonaga R. (2020). SGLT2 inhibitor ipragliflozin attenuates breast cancer cell proliferation. Endocr. J..

[2] Scafoglio C., Hirayama B.A., Kepe V., Liu J., Ghezzi C., Satyamurthy N., Moatamed N.A., Huang J., Koepsell H., Barrio J.R. (2015). Functional expression of sodium-glucose transporters in cancer. Proc. Natl. Acad. Sci. USA.

[3] Shiba K., Tsuchiya K., Komiya C., Miyachi Y., Mori K., Shimazu N., Yamaguchi S., Ogasawara N., Katoh M., Itoh M. (2018). Canagliflozin, an SGLT2 inhibitor, attenuates the development of hepatocellular carcinoma in a mouse model of human NASH. Sci. Rep..

[4] Saito T., Okada S., Yamada E., Shimoda Y., Osaki A., Tagaya Y., Shibusawa R., Okada J., Yamada M. (2015). Effect of dapagliflozin on colon cancer cell. Endocr. J..

[5] Tang H., Dai Q., Shi W., Zhai S., Song Y., Han J. (2017). SGLT2 inhibitors and risk of cancer in type 2 diabetes: A systematic review and meta-analysis of randomised controlled trials. Diabetologia.

[6] Kaji K., Nishimura N., Seki K., Sato S., Saikawa S., Nakanishi K., Furukawa M., Kawaratani H., Kitade M., Moriya K. (2018). Sodium glucose cotransporter 2 inhibitor canagliflozin attenuates liver cancer cell growth and angiogenic activity by inhibiting glucose uptake. Int. J. Cancer.

[7] Villani L.A., Smith B.K., Marcinko K., Ford R.J., Broadfield L.A., Green A.E., Houde V.P., Muti P., Tsakiridis T., Steinberg G.R. (2016). The diabetes medication Canagliflozin reduces cancer cell proliferation by inhibiting mitochondrial complex-I supported respiration. Mol. Metab..

[8] Obara K., Shirakami Y., Maruta A., Ideta T., Miyazaki T., Kochi T., Sakai H., Tanaka T., Seishima M., Shimizu M. (2017). Preventive effects of the sodium glucose cotransporter 2 inhibitor tofogliflozin on diethylnitrosamine-induced liver tumorigenesis in obese and diabetic mice. Oncotarget.

[9] Kuang H., Liao L., Chen H., Kang Q., Shu X., Wang Y. (2017). Therapeutic effect of sodium glucose co-transporter 2 inhibitor dapagliflozin on renal cell carcinoma. Med. Sci. Monit..

[10] Scafoglio C.R., Villegas B., Abdelhady G., Bailey S.T., Liu J., Shirali A.S., Wallace W.D., Magyar C.E., Grogan T.R., Elashoff D. (2018). Sodium-glucose transporter 2 is a diagnostic and therapeutic target for early-stage lung adenocarcinoma. Sci. Transl. Med..

[11] Nasiri A.R., Rodrigues M.R., Li Z., Leitner B.P., Perry R.J. (2019). SGLT2 inhibition slows tumor growth in mice by reversing hyperinsulinemia. Cancer Metab..

[12] Papadopoli D., Uchenunu O., Palia R., Chekkal N., Hulea L., Topisirovic I., Pollak M., St-Pierre J. (2021). Perturbations of cancer cell metabolism by the antidiabetic drug canagliflozin. Neoplasia.

[13] Abdul-Ghani M., Stefano Del Prato S., Chilton R., DeFronzo R.A. (2016). SGLT2 Inhibitors and Cardiovascular Risk: Lessons Learned From the EMPA-REG OUTCOME Study. Diabetes Care.

[14] Sanjay K. (2016). Sodium-Glucose Cotransporter 2 (SGLT2) Inhibitors and cardiovascular Disease: A Systematic Review. Cardiol. Ther..

[15] Tamargo J. (2019). Sodium–glucose Cotransporter 2 Inhibitors in Heart Failure: Potential Mechanisms of Action, Adverse Effects and Future Developments. Eur. Cardiol. Rev..

[16] Packer M. (2019). Lessons learned from the DAPA-HF trial concerning the mechanisms of benefit of SGLT2 inhibitors on heart failure events in the context of other large-scale trials nearing completion. Cardiovasc. Diabetol..

[17] Mahaffey K.W., Neal B., Perkovic V., de Zeeuw D., Fulcher G., Erondu N., Shaw W., Fabbrini E., Sun T., Li Q. (2018). Canagliflozin for Primary and Secondary Prevention of Cardiovascular Events Results from the CANVAS Program (Canagliflozin Cardiovascular Assessment Study). Circulation.

[18] Rådholm K., Figtree G., Perkovic V., Solomon S.D., Mahaffey K.W., de Zeeuw D., Fulcher G., Barrett T.D., Shaw W., Desai M. (2018). Canagliflozin and Heart Failure in Type 2 Diabetes Mellitus Results from the CANVAS Program. Circulation.

[19] Shah S.R., Najim N.I., Abbasi Z., Fatima M., Jangda A.A., Shahnawaz W., Shahid M., Shah S.A. (2018). Canagliflozin and Cardiovascular disease—results of the CANVAS trial. J. Community Hosp. Intern. Med. Perspect..

[20] Sezai A., Sekino H., Unosawa S., Taoka M., Osaka S., Tanaka M. (2019). Canagliflozin for Japanese patients with chronic heart failure and type II. Cardiovasc. Diabetol..

[21] Brown E., Heerspink H.J.L., Cuthbertson D.J., Wilding J.P.H. (2021). SGLT2 inhibitors and GLP-1 receptor agonists: Established and emerging indications. Lancet.

[22] Cherney D., Dagogo-Jack S., McGuire D.K., Cosentino F., Pratley R., Shih W.J., Frederich R., Maldonado M., Liu J., Wang S. (2021). Kidney outcomes using a sustained ≥40% decline in eGFR: A meta-analysis of SGLT2 inhibitor trials. Clin. Cardiol..

[23] Heston T.F., Olson A.H., Randall N.R. (2017). Canagliflozin lowers blood sugar, but does it also lower cardiovascular risk? Maybe not. Ann. Transl. Med..

[24] Kondo H., Takahashi N. (2019). Reduced hospitalization for heart failure using anti-diabetic drug dapagliflozin: Implications of DECLARE–TIMI 58 for the basic science community. Cardiovasc. Res..

[25] Zinman B., Wanner C., Lachin J.M., Fitchett D., Bluhmki E., Hantel S., Mattheus M., Devins T., Johansen O.E., Woerle H.J. (2015). Empagliflozin, cardiovascular outcomes, and mortality in type 2 diabetes. N. Engl. J. Med..

[26] Butler J., Hamo C.E., Filippatos G., Pocock S.J., Bernstein R.A., Brueckmann M., Cheung A.K., George J.T., Green J.B., Januzzi J.L. (2017). The potential role and rationale for treatmentof heart failure with sodium–glucose co-transporter 2 inhibitors. Eur. J. Heart Fail..

[27] Maejima Y. (2019). SGLT2 Inhibitors Play a Salutary Role in Heart Failure via Modulation of the Mitochondrial Function. Front. Cardiovasc. Med..

[28] Dutka M., Bobiński R., Ulman-Włodarz I., Hajduga M., Bujok J., Pająk C., Ćwiertnia M. (2021). Sodium glucose cotransporter 2 inhibitors: Mechanisms of action in heart failure. Heart Fail. Rev..

[29] Nakano D., Kawaguchi T., Iwamoto H., Hayakawa M., Koga H., Torimura T. (2020). Effects of canagliflozin on growth and metabolic reprograming in hepatocellular carcinoma cells: Multi-omnics analysis of metabolomics and absolute quantification proteomics (iMPAQT). PLoS ONE.

[30] Madunić I.V., Madunić J., Breljak D., Karaica D., Sabolić I. (2018). Sodium-glucose cotransporters: New targets of cancer therapy?. Arh. Hig. Rada Toksikol..

[31] Vrhovac I., Breljak D., Sabolić I. (2014). Glucose transporters in the mammalian blood cells. Period. Biol..

[32] Mueckler M., Thorens B. (2013). The SLC2 (GLUT) family of membrane transporters. Mol. Asp. Med..

[33] Wright E. (2013). Glucose transport families SLC5 and SLC50. Mol. Asp. Med..

[34] Wright E., Loo D., Hirayama B. (2011). Biology of human sodium glucose transporters. Physiol. Rev..

[35] Hediger M., Rhoads D. (1994). Molecular physiology of sodiumglucose cotransporters. Physiol. Rev..

[36] Chao E.C., Henry R.R. (2010). SGLT2 inhibition—A novel strategy for diabetes treatment. Nat. Rev. Drug Discov..

[37] Bhartia M., Tahrani A.A., Barnett A.H. (2011). SGLT-2 inhibitors in development for type 2 diabetes treatment. Rev. Diabet. Stud..

[38] Ferrannini E., Solini A. (2012). SGLT2 inhibition in diabetes mellitus: Rationale and clinical prospects. Nat. Rev. Endocrinol..

[39] Wright E.M., Turk E. (2004). The sodium/glucose cotransport family SLC5. Pflug. Arch..

[40] Perry R.J., Shulman G.I. (2020). Sodium-glucose cotransporter-2 inhibitors: Understanding the mechanisms for therapeutic promise and persisting risks. J. Biol. Chem..

[41] Sabolic I., Vrhovac I., Eror D.B., Gerasimova M., Rose M., Breljak D., Ljubojevic M., Brzica H., Sebastiani A., Thal S.C. (2012). Expression of Na^+^-D-glucose cotransporter SGLT2 in rodents is kidneyspecific and exhibits sex and species differences. Am. J. Physiol.-Cell Physiol..

[42] Vrhovac I., Balen Eror D., Klessen D., Burger C., Breljak D., Kraus O., Radović N., Jadrijević S., Aleksic I., Walles T. (2015). Localizations of Na^+^-Dglucose cotransporters SGLT1 and SGLT2 in human kidney and of SGLT1 in human small intestine, liver, lung, and heart. Pflügers Arch..

[43] Madunić I.V., Breljak D., Karaica D., Koepsell H., Sabolić I. (2017). Expression profiling and immunolocalization of Na^+^-D-glucose-cotransporter 1 in mice employing knockout mice as specificity control indicate novel locations and differences between mice and rats. Pflügers Arch..

[44] Chen J., Williams S., Ho S., Loraine H., Hagan D., Whaley J.M., Feder J.N. (2010). Quantitative PCR tissue expression profiling of the human SGLT2 gene and related family members. Diabetes Ther..

[45] Kashiwagi Y. (2015). Expression of SGLT1 in human hearts and impairment of cardiac glucose uptake by phlorizin during ischemiareperfusion injury in mice. PLoS ONE.

[46] Wright E.M. (2001). Renal Na^+^-glucose cotransporters. Am. J. Physiol. Renal Physiol..

[47] Zhou J., Zhu J., Yu S.J., Ma H.L., Chen J., Ding X.F., Chen G., Liang Y., Zhang Q. (2020). Sodium-glucose co-transporter-2 (SGLT-2) inhibition reduces glucose uptake to induce breast cancer cell growth arrest through AMPK/mTOR pathway. Biomed. Pharmacother..

[48] Ishikawa N., Oguri T., Isobe T., Fujitaka K., Kohno N. (2001). SGLT gene expression in primary lung cancers and their metastatic lesions. Jpn. J. Cancer Res..

[49] Zhang X., Zhang X., Liu X., Qi P., Wang H., Ma Z., Chai Y. (2019). MicroRNA-296, a suppressor non-coding RNA, downregulates SGLT2 expression in lung cancer. Int. J. Oncol..

[50] Billger M., Kirk J., Chang J., Bédard A., Attalla B., Haile S., Söderberg M. (2019). A study in a rat initiation-promotion bladder tumour model demonstrated no promoter/progressor potential of dapagliflozin. Regul. Toxicol. Pharmacol..

[51] Yamamoto L., Yamashita S., Nomiyama T., Kawanami T., Hamaguchi Y., Shigeoka T., Horikawa T., Tanaka Y., Yanase T., Kawanami D. (2021). Sodium-glucose cotransporter 2 inhibitor canagliflozin attenuates lung cancer cell proliferation in vitro. Diabetol. Int..

[52] Koepsell H. (2017). The Na^+^ -D-glucose cotransporters SGLT1 and SGLT2 are targets for the treatment of diabetes and cancer. Pharmacol. Ther..

[53] Packer M., Anker S.D., Butler J., Filippatos G., Pocock S.J., Carson P., Januzzi J., Verma S., Tsutsui H., Brueckmann M. (2020). Cardiovascular and Renal Outcomes with Empagliflozin in Heart Failure. N. Engl. J. Med..

[54] Anker S.D., Butler J., Filippatos G., Ferreira J.P., Bocchi E., Böhm M., Brunner-La Rocca H.P., Choi D.J., Chopra V., Chuquiure-Valenzuela E. (2021). Empagliflozin in Heart Failure with a Preserved Ejection Fraction. N. Engl. J. Med..

[55] Yang X.Q., Xu C., Sun Y., Han R.F. (2013). Diabetes mellitus increases the risk of bladder cancer: An updated meta-analysis. Asian Pac. J. Cancer Prev..

[56] Fang H., Yao B., Yan Y., Xu H., Liu Y., Tang H., Zhou J., Cao L., Wang W., Zhang J. (2013). Diabetes mellitus increases the risk of bladder cancer: An updated meta-analysis of observational studies. Diabetes Technol. Ther..

[57] Zhu Z., Wang X., Shen Z., Lu Y., Zhong S., Xu C. (2013). Risk of bladder cancer in patients with diabetes mellitus: An updated meta-analysis of 36 observational studies. BMC Cancer.

[58] Tseng C.H. (2011). Diabetes and risk of bladder cancer: A study using the National Health Insurance database in Taiwan. Diabetologia.

[59] Dąbrowski M. (2021). Diabetes, Antidiabetic Medications and Cancer Risk in Type 2 Diabetes: Focus on SGLT-2 Inhibitors. Int. J. Mol. Sci..

[60] Shi N., Shi Y., Xu J., Si Y., Yang T., Zhang M., Ng D.M., Li X., Xie F. (2021). SGLT-2i and Risk of Malignancy in Type 2 Diabetes: A Meta-Analysis of Randomized Controlled Trials. Front. Public Health.

[61] Carstensen B., Jørgensen M.E., Friis S. (2014). The epidemiology of diabetes and cancer. Curr. Diabetes Rep..

[62] Johnson J.A., Carstensen B., Witte D., Bowker S.L., Lipscombe L., Renehan A.G., Diabetes and Cancer Research Consortium (2012). Diabetes and cancer (1): Evaluating the temporal relationship between type 2 diabetes and cancer incidence. Diabetologia.

[63] Onitilo A.A., Engel J.M., Glurich I., Stankowski R.V., Williams G.M., Doi S.A. (2012). Diabetes and cancer II: Role of diabetes medications and influence of shared risk factors. Cancer Causes Control.

[64] Tseng C.H. (2012). Pioglitazone and bladder cancer: A population-based study of Taiwanese. Diabetes Care.

[65] Mamtani R., Haynes K., Bilker W.B., Vaughn D.J., Strom B.L., Glanz K., Lewis J.D. (2012). Association Between Longer Therapy with Thiazolidinediones and Risk of Bladder Cancer: A Cohort Study. J. Natl. Cancer Inst..

[66] Azoulay L., Yin H., Filion K.B., Assayag J., Majdan A., Pollak M.N., Suissa S. (2012). The use of pioglitazone and the risk of bladder cancer in people with type 2 diabetes: Nested case-control study. BMJ.

[67] Wei L., Macdonald T.M., Mackenzie I.S. (2013). Pioglitazone and bladder cancer: A propensity score matched cohort study. Br. J. Clin. Pharmacol..

[68] Tseng C.H. (2014). A review on thiazolidinediones and bladder cancer in human studies. J. Environ. Sci. Health C Environ. Carcinog. Ecotoxicol. Rev..

[69] Lewis J.D., Ferrara A., Peng T., Hedderson M., Bilker W.B., Quesenberry C.P., Vaughn D.J., Nessel L., Selby J., Strom B.L. (2011). Risk of bladder cancer among diabetic patients treated with pioglitazone: Interim report of a longitudinal cohort study. Diabetes Care.

[70] Lin H.W., Tseng C.H. (2014). A review on the relationship between SGLT2 inhibitors and cancer. Int. J. Endocrinol..

[71] Suissa M., Yin H., Yu O.H.Y., Wong S.M., Azoulay L. (2021). Sodium-Glucose Cotransporter 2 Inhibitors and the Short-term Risk of Breast Cancer Among Women with Type 2 Diabetes. Diabetes Care.

[72] De Jonghe S., Proctor J., Vinken P., Feyen B., Wynant I., Marien D., Geys H., Mamidi R.N., Johnson M.D. (2014). Carcinogenicity in rats of the SGLT2 inhibitor canagliflozin. Chem. Biol. Interact..

[73] Taub M.E., Ludwig-Schwellinger E., Ishiguro N., Kishimoto W., Yu H., Wagner K., Tweedie D. (2015). Sex-, Species-, and Tissue-Specific Metabolism of Empagliflozin in Male Mouse Kidney Forms an Unstable Hemiacetal Metabolite (M466/2) That Degrades to 4-Hydroxycrotonaldehyde, a Reactive and Cytotoxic Species. Chem. Res. Toxicol..

[74] Prentice D.E., Meikle A.W. (1995). A review of drug-induced Leydig cell hyperplasia and neoplasia in the rat and some comparisons with man. Hum. Exp. Toxicol..

[75] Roe F.J.C. (1989). Relevance for man of the effects of lactose, polyols and other carbohydrates on calcium metabolism seen in rats: A review. Hum. Toxicol..

[76] Dicembrini I., Nreu B., Mannucci E., Monami M. (2019). Sodium-glucose co-transporter-2 (SGLT-2) inhibitors and cancer: A meta-analysis of randomized controlled trials. Diabetes Obes. Metab..

[77] Neal B., Perkovic V., Mahaffey K.W., de Zeeuw D., Fulcher G., Erondu N., Shaw W., Law G., Desai M., Matthews D.R. (2017). Canagliflozin and Cardiovascular and Renal Events in Type 2 Diabetes. N. Engl. J. Med..

[78] Wiviott S.D., Raz I., Bonaca M.P., Mosenzon O., Kato E.T., Cahn A., Silverman M.G., Zelniker T.A., Kuder J.F., Murphy S.A. (2019). Dapagliflozin and Cardiovascular Outcomes in Type 2 Diabetes. N. Engl. J. Med..

[79] Jones D. (2011). Diabetes field cautiously upbeat despite possible setback for leading SGLT2 inhibitor. Nat. Rev. Drug Discov..

[80] Pelletier R., Ng K., Alkabbani W., Labib Y., Mourad N., Gamble J.M. (2020). The association of sodium-glucose cotransporter 2 inhibitors with cancer: An overview of quantitative systematic reviews. Endocrinol. Diabetes Metab..

[81] Ptaszynska A., Cohen S.M., Messing E.M., Reilly T.P., Johnsson E., Johnsson K. (2015). Assessing Bladder Cancer Risk in Type 2 Diabetes Clinical Trials: The Dapagliflozin Drug Development Program as a ‘Case Study’. Diabetes Ther..

[82] Wang J., Yang D.L., Chen Z.Z., Gou B.F. (2016). Associations of body mass index with cancer incidence among populations, genders, and menopausal status: A systematic review and meta-analysis. Cancer Epidemiol..

[83] Sun J.W., Zhao L.G., Yang Y., Ma X., Wang Y.Y., Xiang Y.B. (2015). Obesity and risk of bladder cancer: A dose-response meta-analysis of 15 cohort studies. PLoS ONE.

[84] Renehan A.G., Tyson M., Egger M., Heller R.F., Zwahlen M. (2008). Body-mass index and incidence of cancer: A systematic review and meta-analysis of prospective observational studies. Lancet.

[85] Kinduryte Schorling O., Clark D., Zwiener I., Kaspers S., Lee J., Iliev H. (2020). Pooled safety and tolerability analysis of empagliflozin in patients with type 2 diabetes mellitus. Adv. Ther..

[86] Kohler S., Zeller C., Iliev H., Kaspers S. (2017). Safety and Tolerability of Empagliflozin in Patients with Type 2 Diabetes: Pooled Analysis of Phase I-III Clinical Trials. Adv. Ther..

[87] Benedetti R., Benincasa G., Glass K., Chianese U., Vietri M.T., Congi R., Altucci L., Napoli C. (2022). Effects of novel SGLT2 inhibitors on cancer incidence in hyperglycemic patients: A meta-analysis of randomized clinical trials. Pharmacol. Res..

[88] Okada J., Yamada E., Saito T., Yokoo H., Osaki A., Shimoda Y., Ozawa A., Nakajima Y., Pessin J.E., Okada S. (2020). Dapagliflozin Inhibits Cell Adhesion to Collagen I and IV and Increases Ectodomain Proteolytic Cleavage of DDR1 by Increasing ADAM10 Activity. Molecules.

[89] Hung M.H., Chen Y.L., Chen L.J., Chu P.Y., Hsieh F.S., Tsai M.H., Shih C.T., Chao T.I., Huang C.Y., Chen K.F. (2019). Canagliflozin inhibits growth of hepatocellular carcinoma via blocking glucose-influx-induced β-catenin activation. Cell Death Dis..

[90] Warburg O. (1956). On respiratory impairment in cancer cells. Science.

[91] Koppenol W.H., Bounds P.L., Dang C.V. (2011). Otto Warburg’s contributions to current concepts of cancer metabolism. Nat. Rev. Cancer.

[92] DeBerardinis R.J., Lum J.J., Hatzivassiliou G., Thompson C.B. (2008). The biology of cancer: Metabolic reprogramming fuels cell growth and proliferation. Cell. Metab..

[93] Hsu P.P., Sabatini D.M. (2008). Cancer cell metabolism: Warburg and beyond. Cell.

[94] Denko N.C. (2008). Hypoxia, HIF1 and glucose metabolism in the solid tumour. Nat. Rev. Cancer.

[95] Amann T., Maegdefrau U., Hartmann A., Agaimy A., Marienhagen J., Weiss T.S., Stoeltzing O., Warnecke C., Schölmerich J., Oefner P.J. (2009). GLUT1 expression is increased in hepatocellular carcinoma and promotes tumorigenesis. Am. J. Pathol..

[96] Younes M., Brown R.W., Mody D.R., Fernandez L., Laucirica R. (1995). GLUT1 expression in human breast carcinoma: Correlation with known prognostic markers. Anticancer Res..

[97] Haber R.S., Weiser K.R., Pritsker A., Reder I., Burstein D.E. (1997). GLUT1 glucose transporter expression in benign and malignant thyroid nodules. Thyroid.

[98] Haber R.S., Rathan A., Weiser K.R., Pritsker A., Itzkowitz S.H., Bodian C., Slater G., Weiss A., Burstein D.E. (1998). GLUT1 glucose transporter expression in colorectal carcinoma: A marker for poor prognosis. Cancer.

[99] Tohma T., Okazumi S., Makino H., Cho A., Mochizuki R., Shuto K., Kudo H., Matsubara K., Gunji H., Matsubara H. (2005). Overexpression of glucose transporter 1 in esophageal squamous cell carcinomas: A marker for poor prognosis. Dis. Esophagus.

[100] Thorens B., Sarkar H.K., Kaback H.R., Lodish H.F. (1988). Cloning and functional expression in bacteria of a novel glucose transporter present in liver, intestine, kidney, and beta-pancreatic islet cells. Cell.

[101] Fukumoto H., Seino S., Imura H., Seino Y., Eddy R.L., Fukushima Y., Byers M.G., Shows T.B., Bell G.I. (1988). Sequence, tissue distribution, and chromosomal localization of mRNA encoding a human glucose transporter-like protein. Proc. Natl. Acad. Sci. USA.

[102] Karim S., Adams D.H., Lalor P.F. (2012). Hepatic expression and cellular distribution of the glucose transporter family. World J. Gastroenterol..

[103] Lee S.Y., Jeon H.M., Ju M.K., Kim C.H., Yoon G., Han S.I., Park H.G., Kang H.S. (2012). Wnt/snail signaling regulates cytochrome C oxidase and glucose metabolism. Cancer Res..

[104] Pate K.T., Stringari C., Sprowl-Tanio S., Wang K., TeSlaa T., Hoverter N.P., McQuade M.M., Garner C., Digman M.A., Teitell M.A. (2014). Wnt signaling directs a metabolic program of glycolysis and angiogenesis in colon cancer. EMBO J..

[105] Shibata T., Aburatani H. (2014). Exploration of liver cancer genomes. Nat. Rev. Gastroenterol. Hepatol..

[106] Reya T., Duncan A.W., Ailles L., Domen J., Scherer D.C., Willert K., Hintz L., Nusse R., Weissman I.L. (2003). A role for Wnt signalling in self-renewal of haematopoietic stem cells. Nature.

[107] Zhan T., Rindtorff N., Boutros M. (2016). Wnt signaling in cancer. Oncogene.

[108] Osataphan S., Macchi C., Singhal G., Chimene-Weiss J., Sales V., Kozuka C., Dreyfuss J.M., Pan H., Tangcharoenpaisan Y., Morningstar J. (2019). SGLT2 inhibition reprograms systemic metabolism via FGF21-dependent and -independent mechanisms. JCI Insight.

[109] Yuan H., Han Y., Wang X., Li N., Liu Q., Yin Y., Wang H., Pan L., Li L., Song K. (2020). SETD2 restricts prostate cancer metastasis by integrating EZH2 and AMPK signaling pathways. Cancer Cell.

[110] Leprivier G., Rotblat B. (2020). How does mTOR sense glucose starvation? AMPK is the usual suspect. Cell Death Discov..

[111] Steinberg G.R., Carling D. (2019). AMP-activated protein kinase: The current landscape for drug development. Nat. Rev. Drug Discov..

[112] Carling D., Mayer F.V., Sanders M.J., Gamblin S.J. (2011). AMP-activated protein kinase: Nature’s energy sensor. Nat. Chem. Biol..

[113] Hardie D.G., Carling D. (1997). The AMP-activated protein kinase: Fuel gauge of the mammalian cell. Eur. J. Biochem..

[114] Sanders M.J., Grondin P.O., Hegarty B.D., Snowden M.A., Carling D. (2007). Investigating the mechanism for AMP activation of the AMP-activated protein kinase cascade. Biochem. J..

[115] Xiao B., Sanders M.J., Underwood E., Heath R., Mayer F.V., Carmena D., Jing C., Walker P.A., Eccleston J.F., Haire L.F. (2011). Structure of mammalian AMPK and its regulation by ADP. Nature.

[116] Ross F.A., Jensen T.E., Hardie D.G. (2016). Differential regulation by AMP and ADP of AMPK complexes containing different gamma subunit isoforms. Biochem. J..

[117] Lounis M.A., Bergeron K.F., Burhans M.S., Ntambi J.M., Mounier C. (2017). Oleate activates SREBP-1 signaling activity in SCD1-deficient hepatocytes. Am. J. Physiol. Endocrinol. Metab..

[118] Kahn B.B., Alquier T., Carling D., Hardie D.G. (2005). AMP-activated protein kinase: Ancient energy gauge provides clues to modern understanding of metabolism. Cell Metab..

[119] Lally J.S.V., Ghoshal S., DePeralta D.K., Moaven O., Wei L., Masia R., Erstad D.J., Fujiwara N., Leong V., Houde V.P. (2019). Inhibition of Acetyl-CoA Carboxylase by Phosphorylation or the Inhibitor ND-654 Suppresses Lipogenesis and Hepatocellular Carcinoma. Cell Metab..

[120] Zhao Y., Li M., Yao X., Fei Y., Lin Z., Li Z., Cai K., Zhao Y., Luo Z. (2020). HCAR1/MCT1 regulates tumor ferroptosis through the lactate-mediated AMPK-SCD1 activity and its therapeutic implications. Cell Rep..

[121] Jiang X., Stockwell B.R., Conrad M. (2021). Ferroptosis: Mechanisms, biology, and role in disease. Nat. Rev. Mol. Cell. Biol..

[122] Luis G., Godfroid A., Nishiumi S., Cimino J., Blacher S., Maquoi E., Wery C., Collignon A., Longuespée R., Montero-Ruiz L. (2021). Tumor resistance to ferroptosis driven by Stearoyl-CoA Desaturase-1 (SCD1) in cancer cells and Fatty Acid Biding Protein-4 (FABP4) in tumor microenvironment promote tumor recurrence. Redox Biol..

[123] Yang W.S., Kim K.J., Gaschler M.M., Patel M., Shchepinov M.S., Stockwell B.R. (2016). Peroxidation of polyunsaturated fatty acids by lipoxygenases drives ferroptosis. Proc. Natl. Acad. Sci. USA.

[124] Magtanong L., Ko P.J., To M., Cao J.Y., Forcina G.C., Tarangelo A., Ward C.C., Cho K., Patti G.J., Nomura D.K. (2019). Exogenous monounsaturated fatty acids promote a ferroptosis-resistant Cell State. Cell Chem. Biol..

[125] Tesfay L., Paul B.T., Konstorum A., Deng Z., Cox A.O., Lee J., Furdui C.M., Hegde P., Torti F.M., Torti S.V. (2019). Stearoyl-CoA desaturase 1 protects ovarian cancer cells from ferroptotic cell death. Cancer Res..

[126] Fritz V., Benfodda Z., Rodier G., Henriquet C., Iborra F., Avancès C., Allory Y., de la Taille A., Culine S., Blancou H. (2010). Abrogation of de novo lipogenesis by stearoyl-CoA desaturase 1 inhibition interferes with oncogenic signaling and blocks prostate cancer progression in mice. Mol. Cancer Ther..

[127] Yan H., Li Z., Shen Q., Wang Q., Tian J., Jiang Q., Gao L. (2017). Aberrant expression of cell cycle and material metabolism related genes contributes to hepatocellular carcinoma occurrence. Pathol. Res. Pract..

[128] Liu F., Li H., Chang H., Wang J., Lu J. (2015). Identification of hepatocellular carcinoma-associated hub genes and pathways by integrated microarray analysis. Tumori.

[129] Sanli T., Steinberg G.R., Singh G., Tsakiridis T. (2014). AMP-activated protein kinase (AMPK) beyond metabolism: A novel genomic stress sensor participating in the DNA damage response pathway. Cancer Biol. Ther..

[130] Lee C.W., Wong L.L., Tse E.Y., Liu H.F., Leong V.Y., Lee J.M., Hardie D.G., Ng I.O., Ching Y.P. (2012). AMPK promotes p53 acetylation via phosphorylation and inactivation of SIRT1 in liver cancer cells. Cancer Res..

[131] Kennedy S.P., O’Neill M., Cunningham D., Morris P.G., Toomey S., Blanco-Aparicio C., Martinez S., Pastor J., Eustace A.J., Hennessy B.T. (2020). Preclinical evaluation of a novel triple-acting PIM/PI3K/mTOR inhibitor, IBL-302, in breast cancer. Oncogene.

[132] Xu D., Zhou Y., Xie X., He L., Ding J., Pang S., Shen B., Zhou C. (2020). Inhibitory effects of canagliflozin on pancreatic cancer are mediated via the downregulation of glucose transporter-1 and lactate dehydrogenase A. Int. J. Oncol..

[133] Xie Z., Wang F., Lin L., Duan S., Liu X., Li X., Li T., Xue M., Cheng Y., Ren H. (2020). An SGLT2 inhibitor modulates SHH expression by activating AMPK to inhibit the migration and induce the apoptosis of cervical carcinoma cells. Cancer Lett..

[134] Puts G.S., Leonard M.K., Pamidimukkala N.V., Snyder D.E., Kaetzel D.M. (2018). Nuclear functions of NME proteins. Lab. Investig..

[135] Hindupur S.K., Colombi M., Fuhs S.R., Matter M.S., Guri Y., Adam K., Cornu M., Piscuoglio S., Ng C., Betz C. (2018). The protein histidine phosphatase LHPP is a tumour suppressor. Nature.

[136] Zerbe L.K., Kuchta R.D. (2002). The p58 subunit of human DNA primase is important for primer initiation, elongation, and counting. Biochemistry.

[137] Chen X.Y., Li D.F., Han J.C., Wang B., Dong Z.P., Yu L.N., Pan Z.H., Qu C.J., Chen Y., Sun S. (2017). Reprogramming induced by isoliquiritigenin diminishes melanoma cachexia through mTORC2-AKT-GSK3beta signaling. Oncotarget.

[138] Elstrom R.L., Bauer D.E., Buzzai M., Karnauskas R., Harris M.H., Plas D.R., Zhuang H., Cinalli R.M., Alavi A., Rudin C.M. (2004). Akt stimulates aerobic glycolysis in cancer cells. Cancer Res..

[139] Agani F., Jiang B.H. (2013). Oxygen-independent regulation of HIF-1: Novel involvement of PI3K/AKT/mTOR pathway in cancer. Curr. Cancer Drug Targets.

[140] Polet F., Feron O. (2013). Endothelial cell metabolism and tumour angiogenesis: Glucose and glutamine as essential fuels and lactate as the driving force. J. Intern. Med..

[141] Cruys B., Wong B.W., Kuchnio A., Verdegem D., Cantelmo A.R., Conradi L.C., Vandekeere S., Bouché A., Cornelissen I., Vinckier S. (2016). Glycolytic regulation of cell rearrangement in angiogenesis. Nat. Commun..

[142] De Bock K., Georgiadou M., Schoors S., Kuchnio A., Wong B.W., Cantelmo A.R., Quaegebeur A., Ghesquière B., Cauwenberghs S., Eelen G. (2013). Role of PFKFB3-driven glycolysis in vessel sprouting. Cell.

[143] Bergers G., Benjamin L.E. (2003). Tumorigenesis and the angiogenic switch. Nat. Rev. Cancer.

[144] Lee M.S., Moon E.J., Lee S.W., Kim M.S., Kim K.W., Kim Y.J. (2001). Angiogenic activity of pyruvic acid in in vivo and in vitro angiogenesis models. Cancer Res..

[145] Jung S.Y., Song H.S., Park S.Y., Chung S.H., Kim Y.J. (2011). Pyruvate promotes tumor angiogenesis through HIF-1-dependent PAI-1 expression. Int. J. Oncol..

[146] Kihira Y., Yamano N., Izawa-Ishizawa Y., Ishizawa K., Ikeda Y., Tsuchiya K., Tamaki T., Tomita S. (2011). Basic fibroblast growth factor regulates glucose metabolism through glucose transporter 1 induced by hypoxia-inducible factor-1alpha in adipocytes. Int. J. Biochem. Cell Biol..

[147] Torimura T., Ueno T., Kin M., Harada R., Taniguchi E., Nakamura T., Sakata R., Hashimoto O., Sakamoto M., Kumashiro R. (2004). Overexpression of angiopoietin-1 and angiopoietin-2 in hepatocellular carcinoma. J. Hepatol..

[148] Diaz-Sanchez A., Matilla A., Nuñez O., Lorente R., Fernandez A., Rincón D., Campos R., Bañares R., Clemente G. (2013). Serum angiopoietin-2 level as a predictor of tumor invasiveness in patients with hepatocellular carcinoma. Scand. J. Gastroenterol..

[149] Faillaci F., Marzi L., Critelli R., Milosa F., Schepis F., Turola E., Andreani S., Vandelli G., Bernabucci V., Lei B. (2018). Liver Angiopoietin-2 Is a Key Predictor of De Novo or Recurrent Hepatocellular Cancer After Hepatitis C Virus Direct-Acting Antivirals. Hepatology.

[150] Qin G., Luo M., Chen J., Dang Y., Chen G., Li L., Zeng J., Lu Y., Yang J. (2016). Reciprocal activation between MMP-8 and TGF-β1 stimulates EMT and malignant progression of hepatocellular carcinoma. Cancer Lett..

[151] Lempinen M., Lyytinen I., Nordin A., Tervahartiala T., Mäkisalo H., Sorsa T., Isoniemi H. (2013). Prognostic value of serum MMP-8, -9 and TIMP-1 in patients with hepatocellular carcinoma. Ann. Med..

[152] Wei T., Zhang L.N., Lv Y., Ma X.Y., Zhi L., Liu C., Ma F., Zhang X.F. (2014). Overexpression of platelet-derived growth factor receptor alpha promotes tumor progression and indicates poor prognosis in hepatocellular carcinoma. Oncotarget.

[153] Kawaguchi T., Nakano D., Okamura S., Shimose S., Hayakawa M., Niizeki T., Koga H., Torimura T. (2019). Spontaneous regression of hepatocellular carcinoma with reduction in angiogenesis-related cytokines after treatment with sodium-glucose cotransporter 2 inhibitor in a cirrhotic patient with diabetes mellitus. Hepatol. Res..

[154] Mao W., Zhang J., Komer H., Jiang Y., Ying S. (2019). The emerging role of voltage-gated sodium channels in tumor biology. Front. Oncol..

[155] Liu G., Zhu J., Yu M., Cai C., Zhou Y., Yu M., Fu Z., Gong Y., Yang B., Li Y. (2015). Glutamate dehydrogenase is a novel prognostic marker and predicts metastases in colorectal cancer patients. J. Transl. Med..

[156] Di Conza G., Tsai C.H., Ho P.C. (2019). Fifty shades of alpha-Ketoglutarate on cellular programming. Mol. Cell.

[157] Yang C., Ko B., Hensley C.T., Jiang L., Wasti A.T., Kim J., Sudderth J., Calvaruso M.A., Lumata L., Mitsche M. (2014). Glutamine oxidation maintains the TCA cycle and cell survival during impaired mitochondrial pyruvate transport. Mol. Cell.

[158] Li H., Tong C.W., Leung Y., Wong M.H., To K.K., Leung K.S. (2017). Identification of Clinically Approved Drugs Indacaterol and Canagliflozin for Repurposing to Treat Epidermal Growth Factor Tyrosine Kinase Inhibitor-Resistant Lung Cancer. Front. Oncol..

[159] Kim J., Kundu M., Viollet B., Guan K.L. (2011). AMPK and mTOR Regulate Autophagy through Direct Phosphorylation of Ulk1. Nat. Cell Biol..

[160] Shibutani S.T., Saitoh T., Nowag H., Münz C., Yoshimori T. (2015). Autophagy and Autophagy-Related Proteins in the Immune System. Nat. Immunol..

[161] Sciarretta S., Maejima Y., Zablocki D., Sadoshima J. (2018). The Role of Autophagy in the Heart. Annu. Rev. Physiol..

[162] Motzer R.J., Hutson T.E., Tomczak P., Michaelson M.D., Bukowski R.M., Rixe O., Oudard S., Negrier S., Szczylik C., Kim S.T. (2007). Sunitinib versus Interferon Alfa in Metastatic Renal-Cell Carcinoma. N. Engl. J. Med..

[163] Reichardt P., Kang Y.K., Rutkowski P., Schuette J., Rosen L.S., Seddon B., Yalcin S., Gelderblom H., Williams C.C., Fumagalli E. (2015). Clinical Outcomes of Patients with Advanced Gastrointestinal Stromal Tumors: Safety and Efficacy in a Worldwide Treatment-Use Trial of Sunitinib. Cancer.

[164] Di Lorenzo G., Autorino R., Bruni G., Cartenì G., Ricevuto E., Tudini M., Ficorella C., Romano C., Aieta M., Giordano A. (2009). Cardiovascular Toxicity Following Sunitinib Therapy in Metastatic Renal Cell Carcinoma: A Multicenter Analysis. Ann. Oncol..

[165] Narayan V., Keefe S., Haas N., Wang L., Puzanov I., Putt M., Catino A., Fang J., Agarwal N., Hyman D. (2017). Prospective Evaluation of Sunitinib-Induced Cardiotoxicity in Patients with Metastatic Renal Cell Carcinoma. Clin. Cancer Res..

[166] Ren C., Sun K., Zhang Y., Hu Y., Hu B., Zhao J., He Z., Ding R., Wang W., Liang C. (2021). Sodium-Glucose CoTransporter-2 Inhibitor Empagliflozin Ameliorates Sunitinib-Induced Cardiac Dysfunction via Regulation of AMPK-mTOR Signaling Pathway-Mediated Autophagy. Front. Pharmacol..

[167] Kerkela R., Woulfe K.C., Durand J.B., Vagnozzi R., Kramer D., Chu T.F., Beahm C., Chen M.H., Force T. (2009). Sunitinib-induced Cardiotoxicity Is Mediated by Off-Target Inhibition of AMP-Activated Protein Kinase. Clin. Transl Sci..

[168] Laderoute K., Calaoagan J.M., Madrid P.B., Klon A.E., Ehrlich P.J. (2010). SU11248 (Sunitinib) Directly Inhibits the Activity of Mammalian 5′-AMP-Activated Protein Kinase (AMPK). Cancer Biol. Ther..

[169] Quagliariello V., De Laurentiis M., Rea D., Barbieri A., Monti M.G., Carbone A., Paccone A., Altucci L., Conte M., Canale M.L. (2021). The SGLT-2 inhibitor empagliflozin improves myocardial strain, reduces cardiac fibrosis and pro-inflammatory cytokines in non-diabetic mice treated with doxorubicin. Cardiovasc. Diabetol..

[170] Quagliariello V., Vecchione R., Coppola C., Di Cicco C., De Capua A., Piscopo G., Paciello R., Narciso V., Formisano C., Taglialatela-Scafati O. (2018). Cardioprotective effects of nanoemulsions loaded with anti-inflammatory nutraceuticals against doxorubicin-induced cardiotoxicity. Nutrients.

[171] Mele D., Tocchetti C.G., Pagliaro P., Madonna R., Novo G., Pepe A., Zito C., Maurea N., Spallarossa P. (2016). Pathophysiology of anthracycline cardiotoxicity. J. Cardiovasc. Med..

[172] Bertero E., Roma L.P., Ameri P., Maack C. (2018). Cardiac effects of SGLT2 inhibitors: The sodium hypothesis. Cardiovasc. Res..

[173] Goerg J., Sommerfeld M., Greiner B., Lauer D., Seckin Y., Kulikov A., Ivkin D., Kintscher U., Okovityi S., Kaschina E. (2021). Low-Dose Empagliflozin Improves Systolic Heart Function after Myocardial Infarction in Rats: Regulation of MMP9, NHE1, and SERCA2a. Int. J. Mol. Sci..

[174] Baartscheer A., Schumacher C.A., Wüst R.C., Fiolet J.W., Stienen G.J., Coronel R., Zuurbier C.J. (2017). Empagliflozin decreases myocardial cytoplasmic Na. Diabetologia.

[175] Eliaa S.G., Al-Karmalawy A.A., Saleh R.M., Elshal M.F. (2020). Empagliflozin and Doxorubicin Synergistically Inhibit the Survival of Triple-Negative Breast Cancer Cells via Interfering with the mTOR Pathway and Inhibition of Calmodulin: In Vitro and Molecular Docking Studies. ACS Pharmacol. Transl. Sci..

[176] Zhong J., Sun P., Xu N., Liao M., Xu C., Ding Y., Cai J., Zhang Y., Xie W. (2020). Canagliflozin inhibits p-gp function and early autophagy and improves the sensitivity to the antitumor effect of doxorubicin. Biochem. Pharmacol..

[177] Sahakian N., Cattieuw L., Ramillon-Cury C., Corroller A.B., Silvestre-Aillaud P., Béliard S., Valéro R. (2021). SGLT2 inhibitors as potentially helpful drugs in PI3K inhibitor-induced diabetes: A case report. Clin. Diabetes Endocrinol..

[178] André F., Ciruelos E., Rubovszky G., Campone M., Loibl S., Rugo H.S., Iwata H., Conte P., Mayer I.A., Kaufman B. (2019). Alpelisib for PIK3CA-mutated, hormone receptor-positive advanced breast cancer. N. Engl. J. Med..

[179] Jump C., Abramson V., Wellons M. (2017). Ketoacidosis with canagliflozin prescribed for phosphoinositide 3-kinase inhibitor–induced hyperglycemia: A case report. J. Investig. Med. High Impact Case Rep..

[180] Jump D.B. (2011). Fatty acid regulation of hepatic lipid metabolism. Curr. Opin. Clin. Nutr. Metab. Care.

[181] Angelopoulou A., Kolokithas-Ntoukas A., Papaioannou L., Kakazanis Z., Khoury N., Zoumpourlis V., Papatheodorou S., Kardamakis D., Bakandritsos A., Hatziantoniou S. (2018). Canagliflozin-loaded magnetic nanoparticles as potential treatment of hypoxic tumors in combination with radiotherapy. Nanomedicine.

[182] World Health Organization Cancer Overview. https://www.who.int/health-topics/cancer#tab=tab_1.

